# Effect of intraperitoneal ropivacaine during and after cytoreductive surgery on time-interval to adjuvant chemotherapy in advanced ovarian cancer: a randomised, double-blind phase III trial

**DOI:** 10.1016/j.bja.2024.10.015

**Published:** 2024-11-20

**Authors:** Emma Hasselgren, Nina Groes-Kofoed, Henrik Falconer, Håkan Björne, Diana Zach, Daniel Hunde, Hemming Johansson, Mihaela Asp, Päivi Kannisto, Anil Gupta, Sahar Salehi

**Affiliations:** 1 Department of Physiology and Pharmacology, Division of Anaesthesiology, Karolinska Institutet, Stockholm, Sweden, Department of Perioperative Medicine and Intensive Care, Karolinska University Hospital, Stockholm, Sweden; 2 Department of Women’s and Children’s Health, Division of Obstetrics and Gynaecology, Karolinska Institutet, Stockholm, Sweden, Department of Pelvic Cancer, Theme Cancer, Karolinska University Hospital, Stockholm, Sweden; 3 Department of Oncology-Pathology, Karolinska Institutet, Stockholm, Sweden; 4 Department of Clinical Science, Division of Obstetrics and Gynaecology Lund University, Lund, Sweden, Department of Obstetrics and Gynaecology, Skåne University Hospital, Lund, Sweden

**Keywords:** cytoreductive surgery, intraperitoneal, local anaesthetic, ovarian cancer, ropivacaine, return to intended oncologic therapy, time interval to chemotherapy

## Abstract

**Background:**

In a previous phase II trial, intraperitoneal local anaesthetics shortened the time interval between surgery and adjuvant chemotherapy, an endpoint associated with improved survival in advanced ovarian cancer. Our objective was to test this in a phase III trial.

**Methods:**

A double-blind, phase III parallel superiority trial was conducted at two university hospitals in Sweden, within a public and centralised healthcare system. Women >18 yr with advanced ovarian cancer scheduled for cytoreductive surgery, an ASA physical status of 1–3 with no speech/language issues, were eligible. Participants were randomly assigned using a central computerised system to receive either ropivacaine 0.2% or saline 0.9% (placebo) intraperitoneally during and after surgery. The primary endpoint was time to return to intended oncologic therapy (RIOT), analysed using *t*-test and linear regression adjusted for centre.

**Results:**

Of the 225 women randomised between August 2020 and December 2023 (ropivacaine *n*=113; placebo *n*=112), 175 were included in the modified intention-to-treat analysis (ropivacaine *n*=86; placebo *n*=89). Median age: ropivacaine group 64 yr (56–73 yr), placebo group: 66 yr (57–74 yr). The mean RIOT in the ropivacaine group was 26.5 days *vs* 25.8 days in the placebo group, with a mean difference of 0.7 days (−2.2 to 3.4 days; *P*=0.65). Per-protocol analysis of 166 women yielded similar results, mean difference of 0.5 days (−2.4 to 3.4 days; *P*=0.74) days. There were no differences in short-term recovery or postoperative morbidity.

**Conclusion:**

Intraperitoneal local anaesthetic did not shorten the time to RIOT among women undergoing surgery for advanced ovarian cancer in this trial.

**Clinical trial registration:**

ClinicalTrials.gov (NCT04065009), European Union Clinical Trials Register (2019-003299-38/SE).

**Editor's key points**

• There is widespread interest in new approaches to modifying tumour biology to improve patient outcomes after cancer surgery.
• Local anaesthetic agents administered near to the tumour during surgery are thought to modify the systemic inflammatory response to cancer surgery, improving patient recovery and allowing an earlier return to oncological therapies.
• In this trial intraperitoneal injection of local anaesthetic did not appear to shorten the time to return to oncologic therapy among women undergoing surgery for advanced ovarian cancer.

Standard first-line treatment in epithelial ovarian cancer, an aggressive malignancy with major tumour dissemination to the peritoneal cavity, is surgery and chemotherapy combined. The aim of cytoreductive surgery is to resect gross tumour to enhance the efficacy of adjuvant chemotherapy.^1^^,^^2^ Complete macroscopic surgical resection of tumour followed by adjuvant chemotherapy has repeatedly been associated with the most favourable survival.^3^^,^^4^ Accordingly, the aim of surgery is to achieve complete macroscopic resection, often requiring the removal of large sections of the peritoneum and multiple organs. Such extensive surgery may increase the risk of serious postoperative complications and a subsequent delay in postoperative recovery, potentially leading to postponed or inhibited administration of adjuvant chemotherapy. A longer interval between surgery and return to intended oncologic therapy (RIOT), specifically the start of adjuvant chemotherapy, has been associated with inferior overall survival.^5^, ^6^, ^7^ Consequently, RIOT is a relevant clinical endpoint to consider.

The surgical trauma and general anaesthesia are followed by physiological responses, including an inflammatory cascade which promotes the wound healing process.^8^ Wound healing shares many aspects with the development of cancer including activation of signalling pathways for proliferation and migration, and inflammatory and pro-tumorigenic immune cell recruitment.^9^^,^^10^ Consequently, after general surgery and specifically in oncologic surgery, preventing a major systemic inflammatory response and preserving immuno-surveillance in the perioperative period is fundamental to promote an improved postoperative outcome.^11^^,^^12^

Local anaesthetics (LAs) are known analgesic agents. Additionally, LAs also have anti-inflammatory properties and attenuate mediators involved in the cascade of inflammation following surgical trauma.^13^^,^^14^ Accordingly, it has been suggested that LA administered at the site of injury might promote early postoperative recovery and possibly even oncologic outcome.^15^, ^16^, ^17^ A previous double-blind, randomised, placebo-controlled phase II trial suggested that LA, specifically ropivacaine, administered in the abdominal cavity during and after surgery may reduce the RIOT interval in women with advanced ovarian cancer.^18^ Ropivacaine has been shown to attenuate inflammatory mediators and decrease cell proliferation.^19^ For this reason, our main objective was to determine, in an adequately powered phase III trial, if intraperitoneal LA (IPLA) administered perioperatively reduces RIOT interval in advanced ovarian cancer. This manuscript reports on primary outcome and the short-term follow-up phase of the protocol.

## Methods

### Trial design and setting

The Intraperitoneal Local Anaesthetics in Ovarian Cancer (IPLA-OVCA) trial was a multicentre, randomised, double-blind, placebo-controlled trial to determine the effect of IPLA on RIOT interval in women with advanced ovarian cancer. The trial was conducted in a setting that comprises a public and centralised healthcare system where treatment is available for all citizens, in two tertiary referral centres (Skåne University Hospital, Lund and Karolinska University Hospital, Stockholm) in Sweden. The trial was conducted in compliance with the Declaration of Helsinki and the guidelines for Good Clinical Practice and was overseen and monitored by the Centre for Clinical Cancer Research at Karolinska University Hospital, Stockholm, Sweden. The study was approved by the Swedish Ethical Review Authority (Dnr: 2019–05149), the Swedish Medical Products Agency (Dnr: 5.1-2019-85294). It was registered at ClinicalTrials.gov (nr: NCT04065009, August 2019) and at the European Union Clinical Trials Register (nr: 2019-003299-38/SE, November 2019). All women signed a written consent after oral and written information. The reporting of the trial adheres to the Consolidated Standards of Reporting Trials guidelines.^20^ The full trial protocol is available in the Supplementary material (Supplement 1).

### Participants

Eligible women were those diagnosed with ovarian cancer, stages III and IV, according to the International Federation of Gynaecology and Obstetrics (FIGO),^21^ scheduled for upfront cytoreductive surgery with curative intent, with an American Society of Anesthesiologists (ASA) physical status of 1–3 and age >18 yr.^21^ Exclusion criteria were: contraindication to epidural anaesthesia (to reduce the risk of uneven distribution of systemic levels of LAs attributed by the epidural route), allergy to any component drugs used during epidural or intraperitoneal anaesthesia (sufentanil, ropivacaine), had cognitive, speech or language difficulties, or if cytoreductive surgery was not attempted at time of upfront laparotomy because of extent of disease. Women who received other histopathologic diagnosis than ovarian cancer at or after surgery, or did not receive adjuvant chemotherapy were excluded after randomisation, before unblinding of assigned intervention.

### Intervention and control/placebo

During surgery 40 ml of study treatment (ropivacaine 0.2% [experimental] or saline 0.9% [control/placebo]) was rinsed in the abdominal cavity at three time points; after incision, halfway through the surgical procedure, and before closure. A plastic multi-port catheter was inserted through the abdominal wall lateral to the surgical incision before closure and placed with the tip in the pelvis and fixed on the skin with dressing, then connected to an infusion pump (CADD-Solis; Smiths Medical, Kista, Sweden) via a bacterial filter. The pump was pre-programmed to inject 10 ml of study drug, every other hour for 72 h after surgery. The intraperitoneal catheter was removed after 72 h, and the total amount of infused study drug was recorded.

### Outcomes

The primary outcome was time (days) from surgery to start of adjuvant chemotherapy, defined as number of days between the date of surgery to the date of first infusion of adjuvant chemotherapy. The referral to the pathologist and medical oncologist was standardised and occurred on the day of surgery on all patients. Moreover, frozen section to confirm the diagnosis was mandatory to reduce the risk of lead time bias. The decision to start adjuvant chemotherapy was at the discretion of the attending medical gynaecologic oncologist. There are no definitive objective variables or formal guidelines for this decision why it is ultimately a subjective one. Secondary outcomes were: postoperative complications within 30 days of surgery, postoperative pain, morbidity, recovery, and overall survival at 3 and 5 yr. Postoperative complications were defined according to the validated Clavien–Dindo classification.^22^ Pain scores were measured with visual analogue scale at rest and in motion, at arrival in the postoperative unit, and the morning of postoperative days 1–3. Drug volume in the epidural infusion was measured at day 0, from end of surgery to 08:00 at postoperative days 1–3. The amount of rescue i.v. opioid was measured day 0 from end of surgery until midnight, atpostoperative days 1–3. Short-term postoperative morbidity was evaluated at postoperative days 3 and 5 with the validated and updated postoperative morbidity survey (POMS) including 10 modules (pulmonary, infectious, renal, gastrointestinal, cardiovascular, neurological, haematological, wound, pain, mobility). Each module is scored yes or no (e.g. if fever is present the infectious module is scored yes [1 point]).^23^^,^^24^ Postoperative quality of recovery was measured at time of inclusion and postoperative day 3 with the validated and in Swedish translated, Quality of Recovery (QoR)-15 questionnaire, which comprises five domains: pain, physical comfort, physical independence, psychological support, and emotional state. The 11-point numerical scale for each item is summarised to a minimum score of 0 (very poor recovery) and a maximum score of 150 (excellent recovery).^25^^,^^26^ Overall survival was defined from the date of randomisation to the date of death (all-cause) or for patients still alive to the date of last clinical follow-up or contact.

### Randomisation and blinding

Women were randomised on the morning of surgery to receive either the intervention (ropivacaine 0.2%) or placebo/control (0.9% saline) by equal allocation, 1:1. The randomisation procedure was pre-stratified for participating centres in blocks of two (permuted block design), web-based, and performed centrally at the clinical trials unit at the Centre for Clinical Cancer Studies, Theme Cancer, Karolinska University Hospital, Stockholm, Sweden by an unblinded dedicated nurse not involved in the study in any other respect. The same unblinded nurse prepared and masked the study treatment. The study treatment was colour- and odour-free and had identical shape and size in both randomisation arms. Neither the participants nor any personnel were aware of which treatment the patient was allocated to.

### Routine anaesthesia and surgery

All women received thoracic epidural and general anaesthesia with standardised perioperative monitoring and management according to local routines at each hospital. Surgery was performed as per clinical routine with a midline laparotomy incision. To ensure the same lead time for all subjects, the referral for histopathologic review of the specimen and to the medical oncologist for adjuvant chemotherapy was standardised. All women were managed in a high-dependency postoperative care unit (HDU) for one or two nights as appropriate, before being transferred to the in-patient surgical ward. An epidural infusion was used for postoperative pain management. Rescue analgesics were administered as needed after surgery when pain was inadequately controlled by epidural analgesia. Perioperative fluid management and blood transfusions were left at the discretion of the attending physician. Standard systemic adjuvant chemotherapy comprised six cycles of carboplatin AUC6 and paclitaxel 175 mg m^−2^. See Supplementary material (Supplement 1) for full description of routine anaesthesia and surgical details.

### Statistical plan and analysis

Statistical assumptions were based on data from the Swedish Quality Registry of Gynaecologic Cancer where the mean time to start of adjuvant chemotherapy was 31 days (standard deviation [sd]=12) and the previous pilot study where the RIOT interval was reduced by 8 days. To detect a reduction in RIOT interval by 5 days (from 31 to 26 days) which was considered clinically meaningful in the experimental group, the study needed to recruit 182 women using a two-sided significance level of 5% and a power of 80%. To compensate for a 20% drop-out rate the sample size was set to 220 subjects.

Descriptive statistics were presented with numbers and proportions, median, and interquartile ranges (IQR) as appropriate. IQR was presented with 25th–75th percentiles. Distributional differences were tested using Fisher's exact test for categorical variables and Mann–Whitney *U*-test for continuous variables. The primary endpoint, time to start of adjuvant chemotherapy, was analysed using both a *t*-test and a linear regression model including the variable treatment. The multivariate linear regression model was adjusted for participating centre (Karolinska University Hospital and Skåne University Hospital). Results were presented as the mean difference in days together with 95% confidence interval (CI). All analyses of primary and secondary outcome were performed according to a modified intention-to-treat principle and as per protocol. All *P*-values were two-sided and a *P*-value <0.05 was considered significant. All statistical analysis was performed using the statistical software Stata version 18 (StataCorp LLC, College Station, TX, USA).

## Results

**Fig 1:**
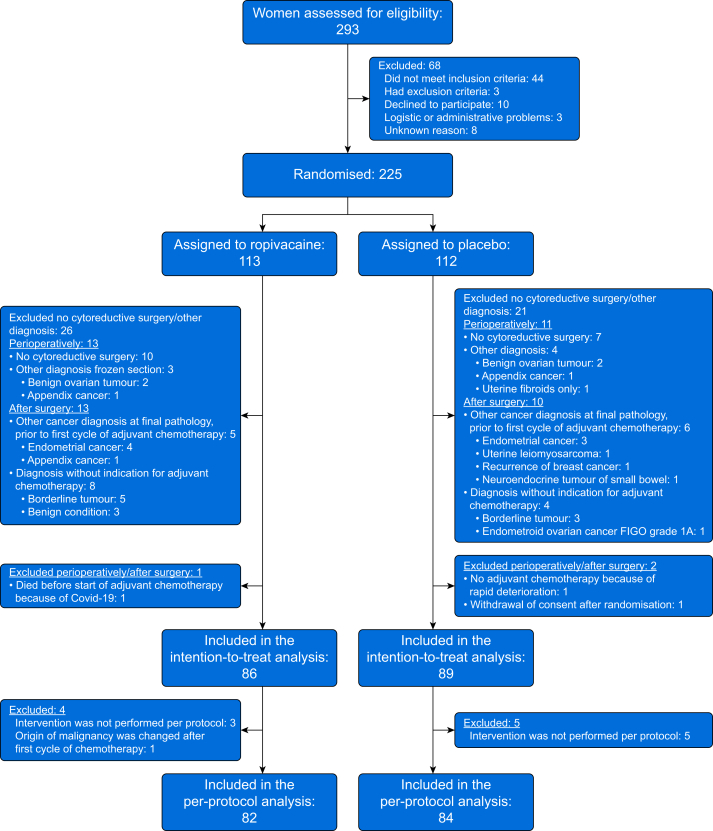
Number of women enrolled, randomly assigned to a treatment group and included in the analyses. FIGO, International Federation of Gynaecology and Obstetrics.

From August 2020 through December 2023, a total of 293 women were assessed for eligibility and 68 were excluded (Fig. 1). A total of 225 patients underwent randomisation, of whom 113 women were assigned to receive ropivacaine, while 112 were assigned to receive placebo (Fig. 1). A total of 86 women in the intervention arm (ropivacaine) and 89 women in the control arm (placebo) were included in the modified intention-to-treat analysis. The corresponding numbers in the per-protocol analysis were 82 and 84, respectively (Fig. 1). A considerable number of patients were excluded after randomisation, owing to the uncertainty of definitive diagnosis, stage, and operability before randomisation. The most prevalent reasons for exclusion after randomisation were perioperatively; no cytoreduction performed (10 in the intervention arm and seven in the control arm) or other diagnosis than ovarian cancer (three in the intervention arm and five in the control arm) and after surgery; diagnosis without indication for adjuvant chemotherapy (eight in the intervention arm and four in the control arm) or other cancer diagnosis at final pathology before first dose of adjuvant chemotherapy (five in the intervention arm and six in the control arm). Three women with advanced ovarian cancer and cytoreductive surgery were excluded after randomisation; two (one in each arm) did not receive adjuvant chemotherapy because of deterioration (i.e. did not have the primary outcome) and one woman in the control group withdrew consent after surgery. There were no distributional differences in the characteristics of participating women by randomisation arms, except that the proportion of women with high-grade serous adenocarcinoma was higher in the intervention arm (ropivacaine) 93% (*n*=80) *vs* 76% (*n*=68) (Table 1). There were no distributional differences in peri- or postoperative variables between randomisation arm (Table 2). Details on the extent of disease and surgical procedures are provided in Supplementary material (Supplement 2).

**Table 1:** Clinical characteristics of randomised women with intention-to-treat. ASA, American Society of Anesthesiologists; BMI, body mass index; CA-125, Cancer-associated antigen 125; ECOG, Eastern Cooperative Oncology Group; FIGO, International Federation of Gynaecology and Obstetrics; IQR, interquartile range. **∗**Two women in the ropivacaine group had missing values. ^†^Age-adjusted Charlson Comorbidity Index, excluding score for ovarian cancer diagnosis. ^‡^Definitive FIGO stage after histopathological result.

Characteristics	Ropivacaine *n*=86	Placebo *n*=89
Age (yr)		
Median (IQR)	64 (56–73)	66 (57–74)
ASA physical status, no. (%)		
1	12 (14)	13 (15)
2	53 (62)	48 (55)
3	21 (24)	27 (31)
ECOG performance status∗, *n* (%)		
0	55 (66)	59 (66)
1	24 (29)	22 (25)
2	5 (6)	8 (9)
Charlson Comorbidity Index^†^		
Median (IQR)	2 (1–3)	2 (1–3)
Plasma albumin (g L^−1^)		
Median (IQR)	35 (33–38)	36 (32–39)
Serum CA-125 (kU L^−1^)		
Median (IQR)	552 (207–1200)	430 (135–1061)
BMI (kg m^−2^)		
Median (IQR)	25 (22–28)	26 (23–29)
Smoking status, *n* (%)		
Yes	8 (9)	4 (5)
No	78 (91)	85 (96)
FIGO stage^‡^, *n* (%)		
IC	2 (2)	3 (3)
IIB	4 (5)	2 (2)
IIIA	2 (2)	3 (3)
IIIB	6 (7)	3 (3)
IIIC	45 (52)	51 (57)
IVA	8 (9)	13 (15)
IVB	19 (22)	14 (16)
Histologic subtype, *n* (%)		
High grade serous adenocarcinoma	80 (93)	68 (76)
Low grade serous adenocarcinoma	3 (4)	6 (7)
Clear cell adenocarcinoma	2 (2)	7 (8)
Endometroid adenocarcinoma	1 (1)	2 (2)
Other subtypes	0	6 (7)
Hospital, *n* (%)		
Karolinska University Hospital	63 (73)	63 (71)
Skåne University Hospital	23 (27)	26 (29)

**Table 2:** Perioperative and postoperative surgical and anaesthesiologic characteristics. IQR, interquartile range; sd, standard deviation; VAS, visual analogue scale. ∗Surgical complexity score according to Mayo Clinic classification. ^†^Two subjects in the ropivacaine group had missing values and correspondingly, two patients in the placebo group.

Variable	Ropivacaine *n*=86	Placebo *n*=89
**Initial volume of ascites at incision (ml)**		
**Median (IQR)**	300 (50–1200)	200 (100–1400)
**Mean (****sd****)**	1026 (1812)	968 (1578)
**Duration of surgery (min)**		
**Median (IQR)**	303 (210–388)	313 (229–381)
**Mean (****sd****)**	305 (111)	312 (118)
**Surgical** complexity score∗		
**Median (IQR)**	7 (5–10)	8 (5–10)
**Mean (****sd****)**	7 (3)	8 (4)
**Surgical** complexity score group, ***n*****(%)**		
**Low (0–3)**	11 (13)	16 (18)
**Medium (4–7)**	35 (41)	27 (30)
**High (**≥**8)**	40 (47)	46 (52)
**Intra-abdominal residual tumour(**cm**)**, ***n*****(%)**		
**0**	50 (58)	63 (71)
**0.1–0.5**	15 (17)	13 (15)
**0.6–1.0**	11 (13)	2 (2)
**>1.0**	10 (12)	11 (12)
**Estimated blood loss during surgery (ml)**		
**Median (IQR)**	850 (480–1300)	800 (500–1400)
**Mean (****sd****)**	995 (701)	1146 (1075)
**Maintenance anaesthesia,*****n*****(%)**^**†**^		
**Sevoflurane**	56 (67)	57 (66)
**Sevoflurane/remifentanil**	27 (32)	26 (30)
**Propofol/remifentanil**	1 (1)	1 (1)
**Other**	0	3 (3)
**Thoracic epidural,*****n*****(%)**		
**Yes**	83 (97)	87 (98)
**No**	3 (3)	2 (2)

In the modified intention-to-treat analysis, the mean number of days from cytoreductive surgery to first infusion of adjuvant chemotherapy was 26.5 days (sd=10.8) in the intervention arm (ropivacaine) and 25.8 days (sd=7.7) in the control arm (placebo), *P*=0.648, with a mean difference adjusted for centre of 0.7 days (95% CI −2.2 to 3.4; *P*=0.648) (Table 3). In the per protocol analysis the corresponding numbers were 26.4 days (sd=10.8) in the intervention arm (ropivacaine) and 25.9 days (sd=7.9) in the control arm (placebo), *P*=0.734, with a mean difference adjusted for centre of 0.5 days (95% CI −2.4 to 3.4; *P*=0.737) (Table 3).

**Table 3:** Effect of intraperitoneal local anaesthetics on time interval to first infusion of chemotherapy. CI, confidence interval; MD, mean difference in days; sd, standard deviation. ∗Student's *t*-test. ^†^Linear regression adjusted for centre.

	Intention-to-treat				Per-protocol			
	Ropivacaine *n*=86	Placebo *n*=89	*P*-value∗	MD^†^ (95% CI) *P*-value	Ropivacaine *n*=82	Placebo *n*=84	*P*-value∗	MD^†^ (95% CI) *P*-value
**Number of days, mean (sd)**	26.5 (10.8)	25.8 (7.7)	0.648	0.7 (−2.2 to 3.4) 0.648	26.4 (10.8)	25.9 (7.9)	0.734	0.5 (−2.4 to 3.4) 0.737

**Fig 2:**
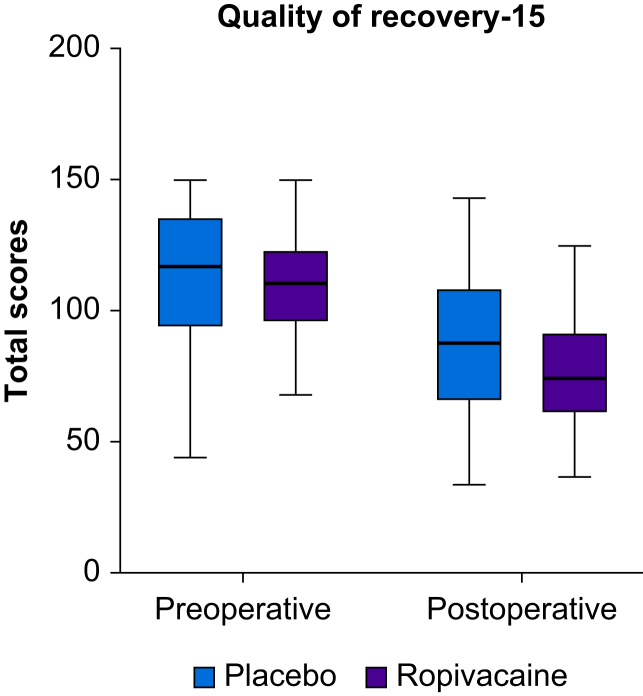
Total quality of recovery score (Quality of recovery-15 questionnaire). Preoperative total score and postoperative day 3 total score. The ends of boxes represent interquartile range, the middle line represents the median, and the whiskers represent minimum and maximum values.

There were no significant differences in postoperative pain, analgesic consumption or postoperative complications within 30 days of surgery (Table 4). The specific complications behind the Clavien–Dindo grading, serious adverse events and volume of study drug are provided in Supplementary material (Supplement 3). No events were assessed as being related to the intervention. Moreover, there were no significant differences in POMS at day 3 or day 5, as shown in the Supplementary material (Supplement 4). The QoR-15 questionnaires were completed in full in 46 patients in each group. The median total preoperative QoR score was 111 (IQR 95–124) in the intervention arm (ropivacaine) *vs* 117 (IQR 93–137) in the control arm (placebo), *P*=0.352. The corresponding postoperative day 3 score was 75 (IQR 60–93) in the intervention arm (ropivacaine) *vs* 88 (IQR 65–110) in the control arm (placebo), *P*=0.103 (Fig. 2).

**Table 4:** Postoperative pain, analgesic consumption or postoperative complications by randomisation. IQR, interquartile range; sd, standard deviation; VAS, visual analogue scale. ∗Mann–Whitney *U*-test if not stated otherwise. ^†^Drugs most used in epidural were either ropivacaine 2 mg ml^−1^ and Sufenta (sufentanil) 0.5 μg ml^−1^ or bupivacaine 1 mg ml^−1^ with addition of either fentanyl 2 μg ml^−1^ or sufentanil 0.5 μg ml^−1^; it was at the discretion of the anaesthesiologist. ^‡^Day 0 - from end of surgery to postoperative day 1 at 08:00, days 1–3 during 24 h from 08:00. ^¶^Ketobemidone, morphine or oxycodone administered i.v. in addition to epidural, Day 0 from end of surgery, days 1–3 from midnight, presented as morphine equivalents. ^§^Measured at 08:00 except for ‘at arrival to postoperative unit’. ^||^Categorised according to Clavien–Dindo classification. ^#^Fisher's exact test. ∗∗Readmission within 30 days after discharge.

Variable	Ropivacaine *n*=86	Placebo *n*=89	*P*-value∗
**Drug volume in epidural^†,‡^ (ml), mean (sd)**			
**Day 0**	78 (27)	77 (22)	0.550
**Day 1**	138 (54)	129 (48)	0.436
**Day 2**	146 (80)	128 (56)	0.296
**Intravenous opioids given^¶^ (mg), mean (sd)**			
**Day 0**	0.6 (2.2)	0.9 (2.6)	0.330
**Day 1**	0.9 (2.8)	1.0 (3.4)	0.737
**Day 2**	0.2 (1.1)	0.3 (1.5)	0.747
**Day 3**	0.4 (2.4)	0.1 (1.1)	0.371
**VAS-score^§^ in rest, median (IQR)**			
**On arrival to postoperative unit**	0 (0–0)	0 (0–2)	0.249
**Day 1**	0 (0–2)	0 (0–3)	0.567
**Day 2**	1 (0–3)	1 (0–3)	0.503
**Day 3**	0 (0–2)	1 (0–2)	0.067
**Postoperative complications within 30 days^||^, *n* (%)**			
≤**II**	61 (71)	60 (67)	0.924#
**III**	23 (27)	26 (30)	
**IVa**	1 (1)	2 (2)	
**IVb**	1 (1)	1 (1)	
**Length of hospital stay (days)**			
**Median (IQR)**	7 (6–9)	8 (6–10)	0.690
**Mean (****sd****)**	9 (7)	9 (7)	
**Readmission∗∗**, ***n*(%)**			
**Yes**	16 (19)	15 (17)	0.844^#^
**No**	70 (81)	73 (83)

## Discussion

In a modified intention-to-treat analysis, this phase III double-blind trial did not demonstrate any effect of intraperitoneal ropivacaine administered perioperatively on the time interval to adjuvant chemotherapy in women with advanced epithelial ovarian cancer subjected to upfront cytoreductive surgery with curative intent.

The purpose of surgical cytoreduction in advanced ovarian cancer is to augment the effect of adjuvant chemotherapy through various mechanisms, including reducing the risk of developing chemotherapy resistance by resection of chemotherapy-resistant tumour clones and improving tumour perfusion.^1^^,^^2^ Moreover, considering the basic concept of tumour kinetics, the prompt initiation of adjuvant chemotherapy after surgical cytoreduction is presumed to yield better outcomes. These theoretic rationales are supported clinically by several observations that suggest an association between shorter RIOT interval and improved survival.^5^, ^6^, ^7^^,^^27^, ^28^, ^29^, ^30^ Consequently, minimising the RIOT interval is of importance for women with advanced ovarian cancer and efforts to reduce this are of significance. A previous randomised, double-blind phase II trial including women with advanced ovarian cancer suggested a significant 8-day reduction in RIOT interval, from 29 to 21 days, after administrations of IPLA.^18^ Positive results of pilot studies might be random. However, they are important for generating plausible hypotheses and must be tested in phase III trials to support changes in clinical practice. For these reasons, we selected RIOT interval as our primary endpoint in the present trial. However, with adequate power we failed to reproduce the findings of the phase II trial.

The mean time to RIOT in both randomisation arms was ∼26 days. This represents a reduction of 5 days in RIOT interval compared with the mean RIOT interval of 31 days used in our power calculation (data from the Swedish Quality Registry of Gynaecologic Cancer). Albeit the precise reason behind this reduction remains uncertain, it is probable that increased clinical attention to the RIOT interval, attributable to the IPLA-OVCA trial, or improved care pathways may have reduced this lead time overall. Moreover, reflect on the fact that the assumptions on which the power and sample size calculations are based on in clinical trials may quickly be outdated with the passing of time. The results furthermore emphasise the importance of conducting phase III trials to define standard care and clinical management.

The anti-inflammatory properties of LA are well known.^13^^,^^14^ Accordingly, LA have the possibility to reduce surgery-induced inflammation with subsequent improvement in postoperative recovery and outcomes.^11^ In a clinical setting, the use of IPLA has been investigated in two previous double-blind phase III trials powered to detect a difference in postoperative recovery as measured by a postoperative recovery scale.^31^^,^^32^ Both trials included patients with colon cancer and instilled ropivacaine in the abdomen during and after surgery and met their primary outcome with an improved short-term postoperative recovery with the addition of IPLA. Moreover, a lower postoperative pain score and reduced use of opioid analgesics was observed in patients allocated to IPLA.^31^^,^^32^

In the present study, with a larger sample size, these findings were not corroborated as there was no difference in reported pain, analgesic consumption or short-term quality of recovery by randomisation arm. It is possible that a higher dose of IPLA could have affected the results. Nevertheless, accounting for the risk of systemic toxicity, when adding the epidural dose of LA, the maximum dose of IPLA was used. Moreover, we may have failed to capture a possible effect by performing the assessment of postoperative recovery at an inaccurate time point (Day 3 after surgery) where patients were still too fatigued from surgery, reflected in the low response rate. There was no difference in short-term postoperative morbidity in the present trial. Similar to previous findings after major abdominal surgery, the prevalence of postoperative morbidity according to POMS was very high on Day 3 and improved on Day 5.^33^ It is also noteworthy that only one subject in each randomisation arm did not receive adjuvant chemotherapy because of postoperative deterioration. The potential impact of LA on oncologic outcomes remains a subject of ongoing research and the vast majority of results from *in vitro* studies overwhelmingly indicate an effect of LA on cancer cells. Several mechanisms are believed to be involved including antiproliferative, antimetastatic, and pro-apoptotic actions.^34^, ^35^, ^36^, ^37^, ^38^, ^39^ However, when examining the potential impact of LA on oncologic outcomes, it is challenging to determine its specific effects because of its involvement in multi-modal surgical and anaesthetic procedures and agents. Nevertheless, interesting observations of favourable oncologic outcomes after addition of LA have been suggested.^40^ Notably, a large phase III trial in women undergoing primary upfront surgery for breast cancer demonstrated an improved disease-free survival and overall survival after peritumoural injection of lidocaine before incision.^16^ Whether IPLA influences oncologic outcome in women with advanced ovarian cancer remains to be investigated after longer follow-up in the present trial.

The target sample size was not met, as a result of a higher-than-expected dropout rate, which is a limitation of our study. This dropout was primarily driven by a larger number of women with diagnoses other than advanced ovarian cancer, exemplifying the known difficulties of accurate perioperative staging when peritoneal carcinomatosis is present. In addition, the trial was conducted during the COVID-19 pandemic, and one can speculate that this might have affected the accrual rate and increased the number of patients with tumour extent incompatible with cytoreductive surgery as a result of patients' delay. Nevertheless, given the clear negative result, it is not plausible that a few additional women would alter any outcome. To reduce risk of selection bias, all randomised subjects in a phase III trial must be included in the intention-to-treat analysis. Nevertheless, conducting clinical trials in this patient group is challenging mainly because of the uncertainty of the definitive diagnosis, stage and operability before the surgical procedure and final histopathology. Moreover, when the primary outcome is a continuous variable and not met (i.e. the patient did not receive adjuvant chemotherapy for whatever reason), these patients may not be included in the analysis. Ideally, all these variables would be known *before* randomisation to avoid exclusion *after* randomisation. However, this was not possible in the present trial as: (1) the study treatment was administered perioperatively including immediately after incision; (2) the definitive diagnosis is set at final pathology (frozen section during surgery is not definitive). We therefore report a modified intention-to-treat analysis, and a per protocol analysis, to ensure these possible sources of bias are clear.

In summary, administration of intraperitoneal local anaesthetic during and after upfront cytoreductive surgery in advanced ovarian cancer did not reduce the time interval to adjuvant chemotherapy in this trial. In addition, they did not improve short-term postoperative recovery or morbidity. There was no evident reduction in postoperative analgesic consumption or pain.

## Authors’ contributions

Contributed to the idea, hypothesis, and study design: AG, EH, SS

Contributed to data acquisition: EH, NGK, HF, HB, DZ, DH, MA, PK, SS

Assembled the data and performed the statistical analysis: EH, NGK, HJ

Interpreted the data and prepared the first draft of the manuscript: EH, NGK, SS

Commented on, edited, reviewed and finally approved the last version of the manuscript: all authors

## Acknowledgements

We would like to thank The Clinical Trials Office and the Centre for Clinical Cancer Research at Karolinska University Hospital, Stockholm Sweden and the research unit at the Department of Perioperative Medicine and Intensive Care at Karolinska University Hospital, Stockholm, Sweden. Moreover, we would like to thank all participating women and hospital staff at Karolinska University Hospital, Stockholm and Skåne University Hospital, Lund, Sweden who contributed to this study. In particular we would like to thank, research nurses Anna Granström, Anna Schenning, Jenny Johansson, and Lovisa Johansson, chief nurse at the in-patient ward of surgical gynaecologic oncology at Karolinska University Hospital, Stockholm Sweden.

## Declaration of interest

The authors declare that they have no conflicts of interest.

## Funding

The Swedish Cancer Society (20 0245 P 03H), Region Stockholm County Council (20200004), the Cancer Research Funds of Radiumhemmet, Stockholm (194142), and the Swedish Society of Medicine (972109). European Society of Anaesthesiology, Research Support Grant (ESAIC_GR_2020_EH).
