# Supplementary material for: Effect of intraperitoneal ropivacaine during and after cytoreductive surgery on time-interval to adjuvant chemotherapy in advanced ovarian cancer: a randomised, double-blind phase III trial
Source: Br J Anaesth. 2024 Nov 20;134(3):662–70. doi: 10.1016/j.bja.2024.10.015 (PMC11867074; doi:10.1016/j.bja.2024.10.015)
Supplement: Multimedia component 1 [file mmc1.pdf]

The IPLA-OVCA trial

Intra-Peritoneal Local Anesthetics in Ovarian Cancer

A randomized double blind, multicenter trial to assess time-interval between cytoreductive surgery and adjuvant chemotherapy after administration of local anesthetic intraperitoneally/perioperatively in advanced epithelial ovarian cancer

Protocol identification number: IPLA-OVCA Version 3.2

Coordinating Investigator/Sponsor
Sahar Salehi MD. Assistant Professor
Department of Pelvic Cancer, Theme Cancer, Karolinska University Hospital, Stockholm Sweden

ClinicalTrials.gov: NCT04065009
Product: Ropivacaine
EudraCT number: 2019-003299-38

Date: Jan 28th, 2021

Authors: Emma Hasselgren, Sahar Salehi

Confidentiality: *This document contains confidential information that must not be disclosed to anyone other than the Investigator Team and members of the Ethics Committees, unless authorized to do so.*

CONTACT INFORMATION	
**Sponsor and Coordinating investigator**	**Sahar Salehi**, MD, PhD, Karolinska University Hospital, Stockholm, Sweden<br>Email: sahar.salehi@sll.se<br>Phone: +46 8 517 70 000
**Trial Steering Committee**	**Emma Hasselgren**, MD, PhD Student, Karolinska University Hospital, Stockholm, Sweden<br>**Diana Zach,** MD, Karolinska University Hospital, Stockholm, Sweden<br>**Henrik Falconer**, MD, Associate Professor, Karolinska University Hospital, Stockholm, Sweden<br>**Håkan Björne**, MD, PhD, Karolinska University Hospital, Stockholm, Sweden<br>**Sahar Salehi**, MD, PhD, Karolinska University Hospital, Stockholm, Sweden
**Sub-Committee on Translational research**	**Sahar Salehi** (Chair), MD, PhD, Karolinska University Hospital, Stockholm, Sweden<br>**Kaisa Lehti,** Associate Professor, Department of Microbiology Tumor and Cell Biology, Karolinska Institutet, Stockholm, Sweden
**Statistician**	**Hemming Johansson**, Biostatistician, Clinical Trials Office, Karolinska University Hospital, Stockholm, Sweden
**Data manager and monitor**	**Claudia Maes,** Datamanager<br>**Katja Tobin**, Clinical Research Manager,<br>**Katja Wiklund**, Clinical Research Manager,<br>CTO, Centre for Clinical Cancer studies, Theme Cancer, Karolinska University Hospital, Stockholm, Sweden

SYNOPSIS

**Protocol title**	**The IPLA-OVCA trial - Intra-Peritoneal Local Anesthetics in Ovarian Cancer**
**EudraCT number**	2019-003299-38
**Primary objective**	To investigate whether Ropivacaine 0.2% administered intraperitoneally during surgery and intermittently thereafter for up to 72 h in patients with stage III-IV epithelial ovarian cancer undergoing cytoreductive surgery leads to earlier start of adjuvant chemotherapy.
**Secondary objectives**	To evaluate postoperative complications and morbidity, quality of recovery and overall survival.
**Study Subjects**	Women with advanced epithelial ovarian cancer stage III-IV scheduled for upfront cytoreductive surgery.
**Study design**	Prospective, randomized placebo controlled double-blinded trial.
**Planned sample size**	220 women
**Inclusion criteria**	• Women > 18 years<br>• ASA I-III<br>• Scheduled for upfront cytoreductive surgery for stage III or IV epithelial ovarian cancer<br>• Signed written informed consent
**Exclusion criteria**	• Contraindication to epidural anesthesia<br>• Allergy to any component drugs used during epidural or intraperitoneal anesthesia (Ropivacaine, Sufentanil)<br>• Uncontrolled renal, liver, heart failure or ischemic heart disease<br>• Speech, language or cognitive difficulties<br>• Women in whom cytoreductive surgery is not attempted at time of upfront laparotomy due to extent of disease

**Test product**	Local anesthetic, Ropivacaine 0.2%
**Administration and Dose**	**Group IPLA**: Intraperitoneal 0.2% Ropivacaine (2 mg/ml)<br>**Group Placebo**: Intraperitoneal Saline
**Primary Outcome**	Time from surgery to start of chemotherapy (days)
**Secondary outcome**	• Post-operative complications<br>• Post-operative morbidity<br>• Quality of recovery<br>• Cardiac and renal impairment<br>• Overall survival at 3 and 5-years
**Study timetable**	First Subject In: **Q3 2020**<br>Last Subject In: **Q3 2023**

INVESTIGATIONAL MEDICINAL PRODUCT (IMP)

Test Product:	Ropivacaine
Pharmaceutical Form:	Solution for infusion
Route of Administration:	Administered intraperitoneally during and for 72 h after surgery.
Dose:	During surgery: 240 mg<br>Postoperatively: 720 mg during 72 hours<br>Total dose (maximal): 960 mg

Signature Page

Investigator's Statement

I, the undersigned, have read and understand the protocol and certify that it contains all necessary information for conducting the study.

I agree to conduct the study according to this protocol and according to the ethical principles that have their origin in the Declaration of Helsinki and that are consistent with ICH-GCP and the applicable national laws and regulations.

Sponsor and Coordinating Investigator:

Sahar Salehi MD. PhD
Department of Pelvic Cancer, Theme Cancer, Karolinska University Hospital, Stockholm

____ ________________

Signature Date

Principal Investigator:

Name: ____________________________________

____ ________________

Signature Date

LIST OF ABBREVIATIONS

Abbreviation	Explanation
AE	Adverse Event
ADR	Adverse Drug Reaction
ARF	Acute renal failure
ASA	American Society of Anesthesiology
C-D classification	Clavien Dindo classification
CRF	Case Report Form
CRS	Cyto-Reductive surgery
CTC	Circulating Tumor Cells
ECM	Extra Cellular Matrix
EDA	Epidural Analgesia
EQ5D	European Quality of life in 5 Dimensions
EOC	Epithelial Ovarian Cancer
EMT	Epithelial to Mesenchymal Transition
FIGO	International Federation of Gynecology and Obstetrics
GCP	Good Clinical Practice
HIF	Hypoxia Inducing Factor
IL-6	Interleukin-6
IMP	Investigational Medicinal Product
LA	Local anesthetics
REC	Regional Ethics Committee
MPA	Medicinal Product Agency
MMP	Matrix Metallo-Proteinases
NKC	Natural Killer Cells
NRS 0-10	Numeric rating Score 0-10
NTproBNP	N-terminal pro b-type natriuretic peptide
PACU	Post-Anesthesia Care Unit
POMS	Postoperative Morbidity Survey
PONV	Postoperative Nausea and Vomiting
QoR15	Quality of Recovery-15
SAE	Serious Adverse Event
SmPC	Summary of Product Characteristics
SUSAR	Suspected Unexpected Serious Adverse Reaction
TGF-b	Tumor Growth factor-beta
TNF-a	Tumor Necrosis factor – alpha
TNM	Tumour, Nodes and Metastases
TnT	Troponin T
VEGF	Vascular Endothelial Growth Factor
VGSC	Voltage Gated Sodium Channels
WHODAS	WHO Disability Assessment Schedule

Table of content

1 Background ... 1
1.1 Epithelial Ovarian cancer ... 1
1.1.1 Treatment of advanced stage EOC ... 2
1.1.2 Time from surgery to start av adjuvant chemotherapy ... 2
1.1.3 Tumorigenesis and the perioperative environment ... 2
1.1.4 Role of local anesthetics and analgesics in cancer metastases ... 3
1.1.5 *In vitro* study on cancer cell proliferation ... 5
1.1.6 Local anesthetics in cytoreductive surgery ... 5
2 Study Objectives ... 6
2.1 Hypothesis ... 6
2.2 Primary Objective ... 6
2.3 Secondary Objectives ... 7
2.4 Primary outcome measure ... 7
2.4.1 Definition of primary outcome measure ... 7
2.5 Secondary outcome measures ... 7
2.5.1 Definition of secondary outcome measures ... 7
3 Study design ... 7
3.1 Study flowchart ... 8
3.2 Schedule of events table ... 9
4 Study enrollment ... 11
4.1 Screening procedure and participant identification ... 11
4.2 Inclusion criteria ... 11
4.3 Exclusion criteria ... 11
4.4 Randomization and registration ... 11
4.5 Blinding and code-breaking ... 12
4.6 Withdrawal criteria ... 12
4.7 End of study ... 13
5 Study treatment ... 13
5.1 Intervention ... 13
5.2 Control ... 13
5.3 Drug preparation ... 13
6 Routine anesthesia and surgery ... 14
6.1 Assessments and Procedures ... 15

7 Translational research component 16
8 Statistical considerations 17
8.1 Primary endpoint 17
8.2 Secondary endpoints 17
8.3 Data analyses 17
8.4 Power and sample size 17
9 Ethical considerations 18
9.1 Risk-Benefit considerations 18
9.2 Institutional review board/Ethics committee 19
9.3 Informed consent and withdrawal 19
9.4 Premature study termination 20
9.5 Patient protection and Good Clinical Practice 20
9.6 Subject identification 20
10 Investigational Medicinal Products 20
10.1 Description of Investigational Medicinal Products 20
10.2 Packaging, Labeling, Storage and Handling of Investigational Medicinal Products 21
10.3 Placebo 21
10.4 Drug accountability 21
10.5 Concomitant Medication 22
10.6 Rescue treatment 22
10.7 Continuation of Treatment 22
11 Assessment of Efficacy and Safety 23
11.1 Baseline assessments 23
11.2 Clinical Efficacy Assessments 23
11.3 Clinical Safety Assessments 23
12 Proceedings for Adverse Events 24
12.1 Definition of Adverse Events 24
12.1.1 Definition of Adverse Reactions 24
12.1.2 Definition of Serious Adverse Events 24
12.1.3 Definition of Suspected Unexpected Serious Adverse Reactions (SUSAR) 24
12.2 Assessment of Adverse Events 25
12.2.1 Assessment of Intensity 25
12.2.2 Assessment of Causality 25
12.3 Methods for Eliciting Adverse Events 25

12.4 Adverse Events **25**
12.4.1 Reporting of Adverse Events (AEs) 25
12.4.2 Reporting of Serious Adverse Events (SAEs) 26
12.4.3 Reporting of Suspected Unexpected Serious Adverse reactions (SUSAR) 26
12.5 Follow-up of Adverse Events **26**
12.6 Follow-up of post-study survival information **26**
12.7 Annual Safety Update (DSUR) **26**
12.8 Safety reference information **27**
13 Significance of study ***27***
14 Administrative considerations ***27***
14.1 Financing **27**
14.2 Publication policy **28**
14.3 Adherence to protocol and protocol amendment **28**
15 Data management and quality control ***28***
15.1 Data source and case report forms **28**
15.2 Data recording and record keeping **29**
15.3 Data protection **29**
15.4 Participant confidentiality **29**
15.5 Storage of study documents **30**
15.6 Quality Control and Monitoring **30**
15.7 Deviations and Violations **31**
15.8 Insurance **31**
16 Appendix ***32***
16.1 Clavien-Dindo **32**
16.2 Post-operative morbidity score (POMS) **32**
16.3 FIGO-staging **34**
16.4 Quality of Recovery, QoR-15 ..... **35**
17 References ***36***

1 Background

1.1 Epithelial Ovarian cancer

Epithelial ovarian cancer (EOC) is the common term for cancer that originate from the surface of the ovary, lining of the fallopian tube and the peritoneum. There are different histopathological subtypes with high grade serous adenocarcinomas being the most prevalent (70%) followed by endometrioid (10%), clear cell (10%), low-grade serous (5%) and mucinous adenocarcinomas (3%).The pattern of dissemination is foremost through early exfoliation or seeding of tumour-cells in the peritoneum (carcinomatosis) and the majority of women have advanced stage disease at presentation, why ovarian cancer is the gynecologic malignancy with highest mortality(1, 2).

Ovarian cancer affects 295 000 women every year globally and causes 185 000 cancer-related deaths annually (3). The highest incidence is seen in Northern Europe and North America and the lowest in South East Asia and parts of Africa(4). In the Nordic countries 2300 women are diagnosed annually and the corresponding number in Sweden is 700 (5, 6). Ovarian cancer constitutes 3% of all female cancers in Sweden and the risk of developing disease before the age of 75 is 1.1% (5). A decline in incidence is evident since the 1970s most probably due to increase in use of contraceptive pills but also change in criteria for diagnosis (an adnexal biopsy is mandatory for diagnosis today). The median age at diagnosis is 63 years.

Risk-factors include genetic mutations, family history, hormone replacement therapy and endometriosis. Pregnancy, lactation, contraceptive pills, sterilization, salpingectomy, hysterectomy and salpingoophorectomy are all factors associated with decreased risk of developing EOC.

Staging of ovarian cancer is surgical and according to the International Federation of Gynecology and Obstetrics (FIGO), latest revised in 2014 (7). In stage III and IV, advanced ovarian cancer, extra-pelvic dissemination is evident. Stage of disease is a strong prognostic factor for survival. The total 5-year relative survival in Sweden is 50% but ranges from 90% for stage I to 20% for stage IV (8). However, treatment is also an important prognostic factor and may increase survival even in advanced stage.

1.1.1 Treatment of advanced stage EOC

Surgery and chemotherapy combined constitute first line treatment. The aim of surgery apart from staging is cytoreduction, i.e. surgical resection of tumour. Radical resection of all tumour visible by the naked eye followed by adjuvant chemotherapy is associated with best chance of prolonged survival(9). However, because of tumour dissemination in the peritoneal cavity, radical surgery is often very extensive with surgery in all quadrants of the abdomen and multi-organ resection with substantial risk of postoperative severe complications and subsequent delay in administration of adjuvant chemotherapy but also perioperative death (10). For these reasons, the surgical team needs to balance risks of extensive surgery versus benefit in prolonged survival on an individual basis, why experience and surgical proficiency is imperative.

1.1.2 Time from surgery to start av adjuvant chemotherapy

The theoretic rational behind radical cytoreductive surgery is to enhance the efficacy of chemotherapy and reducing tumour volume which subsequently increase number of cell divisions needed for tumour regrowth (11-14). It has been suggested that visible post-operative tumour regrowth measured by imaging before start of adjuvant chemotherapy impairs survival (15). Moreover, that longer time-interval to start of adjuvant chemotherapy decreases overall survival (16-18). These findings combined with the theoretic rational behind cytoreductive surgery in advanced EOC implies that the earlier start of adjuvant chemotherapy is beneficial for our patients. The exact cut-off in optimal time-interval between surgery and start of adjuvant chemotherapy has not yet been established. In Sweden, it is generally accepted that adjuvant chemotherapy should commence within 3-4 weeks after surgery and that earlier start is preferable if possible.

1.1.3 Tumorigenesis and the perioperative environment

Surgery, perioperative stress and anesthesia may all modulate the immuno-surveillance mechanisms and compromise host defenses that normally maintain a balance between immunity and tumorigenesis (17, 19). There is increasing evidence that the tissue damage by surgery itself may promote the recurrence and metastases of cancer, which depends largely on the tumor's ability to disseminate and colonize the tissues, combined with the host immunity and inflammatory response. Natural killer (NK) cells act as a primary defense against perioperative cancer metastases (20). Negative effects of anesthetics and analgesics on NK-cell activity may promote pro-inflammatory effects and may also activate cancer cell survival and growth pathways. Additionally, concentrations of tumor-related anti-angiogenic factors are decreased while angiogenic factors such as vascular endothelial growth factors (VEGF-A-D) can be increased (21).

Vascular endothelial growth factor (VEGFA) plays an important role in healthy ovaries by being a regulator of angiogenesis. It is also found in abundance, and together with numerous other growth-regulatory factors, in the ascites of ovarian cancer patients. Collectively these factors play a central role in modulating the tumorigenic microenvironments of ovarian cancer cells. VEGFA has been implicated in the peritoneal dissemination of EOC and the subsequent development and accumulation of malignant ascites (22). VEGFA is over expressed in ovarian tumor cells and is associated with poor prognosis (23). Inhalational anesthetics but not Propofol have been shown to affect VEGFA as well as transforming growth factor-beta (TGF-β) in patients undergoing breast surgery that may affect long-term survival (21). Morphine has been shown to have pro-angiogenic effect, possibly via sensitizing the endothelium to VEGFA and may thereby promote tumor spread in the perioperative period (24). However, opposing theories also prevail. VEGF and TGF-β play a significant role in establishing tumor blood supply and cell proliferation. Therefore, inhibiting the production of VEGF may have beneficial effect by inhibiting tumorigenesis Two randomized trials did not demonstrate any overall survival (OS) benefit with the addition of Bevacizumab, an antibody which targets VEGFA, in patients with advanced EOC (25). However, a select subgroup of patients (sub-optimally debulked, inoperable stage III or stage IV) had significantly improved OS by 4 months (26).

Hypoxia inducing factor (HIF-1) is a transcriptional factor that has also been shown to play an important role for tumor survival (27). Hypoxia in solid tumors leads to activation of HIF-1, which in turn increases cancer cell survival and aggressiveness (28). Paradoxically, hyperoxia may likewise lead to activation of HIF-1 and potentially worsen long-term survival. Other factors such as surgical stress, blood transfusions, extensive wound responses, hypothermia, hyperglycemia, and postoperative pain may also affect immunity and tumor microenvironment. Even after complete excision of the tumor, circulating tumor cells released during the surgical procedure may eventually home to target tissues and lead to recurrence or metastases as they escape the immune and non-immune surveillance (29, 30).

1.1.4 Role of local anesthetics and analgesics in cancer metastases

Preventing a major systemic inflammatory response and preserving immuno-surveillance in the perioperative period are fundamental in promoting improved postoperative outcome in cancer surgery. The severity of injury correlates with the magnitude of inflammation. The potential anti-inflammatory and cytotoxic effects of local anesthetics (LA) such as lidocaine, bupivacain, ropivacaine etc. may effectively reduce postoperative morbidity. *In vitro* studies have shown that amide local anesthetics have cytotoxic activity, which could prove to be beneficial in preventing cancer recurrence (31). Tumor metastases can occur in different ways including transvasation (movement across the the wessel wall), transmigration, intravascular

metastases etc., but the precise mechanism for homing and colonialization of target organs is poorly understood. Because of the surgical stress and the subsequent release of circulating, pro-inflammatory cytokines such as interleukins, TNFa or other related factors during surgery, endothelial barrier function might be impaired by mechanisms such as the activation of the protein tyrosine kinase Src, which can lead to a massive loss of endothelial barrier function and a subsequent increase in vascular permeability (32). Such mechanisms might enhance the extravasation of circulating tumor cells (CTCs) and metastases. *In vitro* studies have demonstrated that the LA Ropivacaine, an amide local anesthetic used commonly, was able to attenuate TNFa-induced signaling events in endothelial cells thus preserving endothelial barrier function and preventing the extravasation of cancer cells (33, 34).

Voltage-gated Sodium Channels (VGSC, Nav) are classically responsible for action potential in excitable cells, but it is also expressed in non-excitable cells including metastatic cancer cells. VGSC is not expressed in a normal way in cancer cells and plays a significant role in disease progression. Highly metastatic ovarian cells showed significantly elevated mRNA expression of Nav 1.2, Nav1.4, Nav1.5 and Nav1.7 (35). Over the past decade, considerable evidence has accumulated that VGSC also contribute to the growth of malignant tissues, particularly in mediating cell migration and metastasis (36). This phenomenon has been demonstrated in malignant cells from various tissues. In prostate and non–small-cell lung cancer, the presence of Nav1.7 channel is a biomarker for the metastatic and invasive potential of the tumor (37, 38) while in ovarian cancer, Nav1.5 is over-expressed compared to non-malignant ovarian cells (39). It is possible that LA was able to prolong survival by blocking VGSC in patients undergoing surgery for malignant melanoma (40).

LA may have a detrimental effect on NK cell activity at very high (and certainly cytotoxic) concentrations *in vitro*. However, a very recent study showed that lidocaine at clinically relevant concentrations of 0.01 and 0.1mM was able to *enhance* NK cytolytic activity *in vitro* through the release of lytic granules (41). In summary, molecular mechanisms by which LA may potentially inhibit metastases of tumor cells are evolving and may differ between individual or different types of tumors. Therefore, clinical studies demonstrating LA efficacy in trials are keenly awaited.

1.1.5 *In vitro* study on cancer cell proliferation

The effects of Lidocaine and Ropivacaine on cell viability and proliferation (primary and metastatic colorectal cancer cell lines, SW480 and SW620) (42) has been studied recently. The authors found that exposure of these colorectal cancer cells to clinically relevant concentration of Ropivacaine (equivalent to approx. 0.2%) decreased cell proliferation (Fig.1).

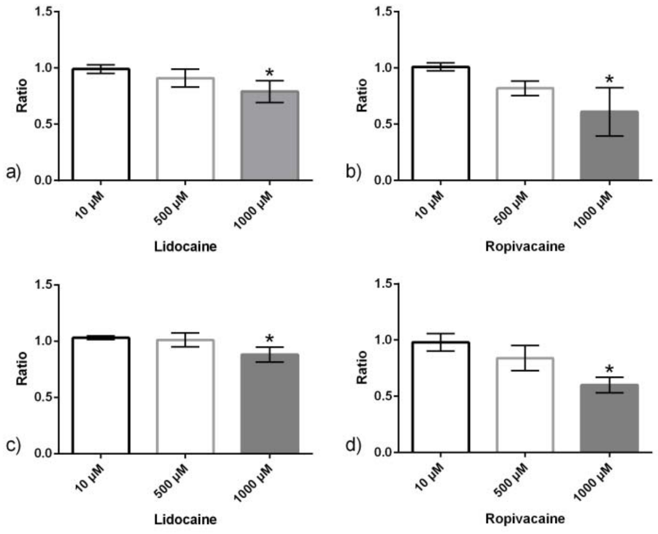

*Figure 1. Cell proliferation. Presented as ratio of PKH67 values between drug exposed cells and unexposed cells (control = 1). a and b) SW480, c and d) SW620. * corrected p < 0.05 after adjustment for multiple comparisons.*

1.1.6 Local anesthetics in cytoreductive surgery

In a recently completed pilot study in 40 patients undergoing CRS surgery for ovarian cancer and randomized and double-blinded to perioperative intraperitoneal local anesthetics (IPLA) or saline (control) during 72 h at Karolinska and Sahlgrenska University Hospitals, we found a striking reduction in the time to start of adjuvant chemotherapy, an important endpoint for overall survival, was significantly lower in the IPLA group, see Table 1 (43).

Table 1. Time interval from surgery to start of chemotherapy by treatment arm, IPLA randomized pilot study

Variable, Median (IQR*)	IPLA (n=20)	Control (n=20)	p-value[1]
**Time-interval surgery to start of chemotherapy, days**	21 (20-29)	30 (21-40)	0.02
**Length of hospital stay, days**	14 (10-17)	12 (9-14)	0.23

*IQR= 25th to 75th percentile. [1]Mann-Whitney U test

In this small study, we did not find any difference in plasma cytokines between the groups (fig 2). A maximum dose of Ropivacaine was administered (epidural and intraperitoneal) during 24 h but plasma concentrations of LA measured after 6, 24 and 48 h remained far below known toxic concentration in humans in this study. Additionally, intraperitoneal LA suppressed the serum cortisol at 6 h, but not at 24 h (results not presented here). We are currently studying 3-5 years' survival in these patients.

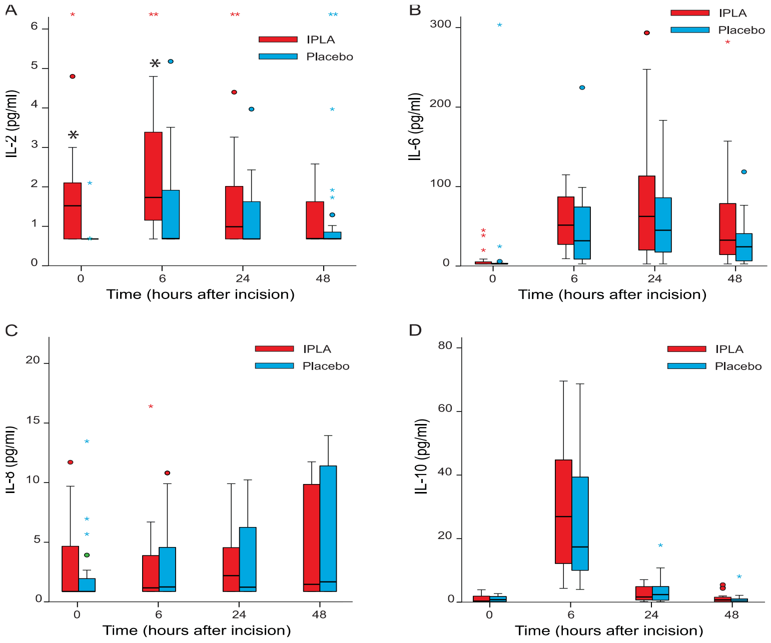

Figure 2. Plasma cytokine concentrations in patients receiving intraperitoneal LA or placebo are shown.

2 Study Objectives

2.1 Hypothesis

Intraperitoneal Ropivacaine administered perioperatively during 72 h shortens the time-interval between surgery and first infusion of adjuvant chemotherapy as compared to placebo.

2.2 Primary Objective

To investigate whether intraperitoneal local anesthetic (Ropivacaine 0.2%) administered during and up to 72 h postoperatively following cytoreductive surgery in patients with advanced stage epithelial ovarian cancer leads to an earlier start of adjuvant chemotherapy as compared to patients receiving placebo (saline).

2.3 Secondary Objectives

Post-operative complications, post-operative morbidity, quality of recovery, cardiac and renal impairment and overall survival.

2.4 Primary outcome measure

Time from surgery to start of adjuvant chemotherapy

2.4.1 Definition of primary outcome measure

Number of days from surgery until first infusion of adjuvant chemotherapy. First day after surgery will count as day one.

2.5 Secondary outcome measures

- Post-operative complications
- Post-operative morbidity
- Quality of recovery
- Cardiac and renal impairment
- Overall survival (OS) at 3- and 5-years

2.5.1 Definition of secondary outcome measures

<u>Post-operative complications</u>: Assessed with Clavien-Dindo classification up to 30 days postoperatively (44).

<u>Post-operative morbidity</u>: Will be assessed by the postoperative morbidity score (POMS) on postoperative day 3 and 5 (45).

<u>Quality of recovery (QoR)</u>: Will be assessed the day before surgery and 3 days after surgery with the QoR-15 questionnaire (46, 47).

<u>Overall survival:</u> Survival time is calculated from the date of randomization to the date of death (all-cause), or for patients still alive to the date of last clinical follow-up or contact.

3 Study design

Prospective, randomized, double-blind, placebo-controlled trial.

3.1 Study flowchart

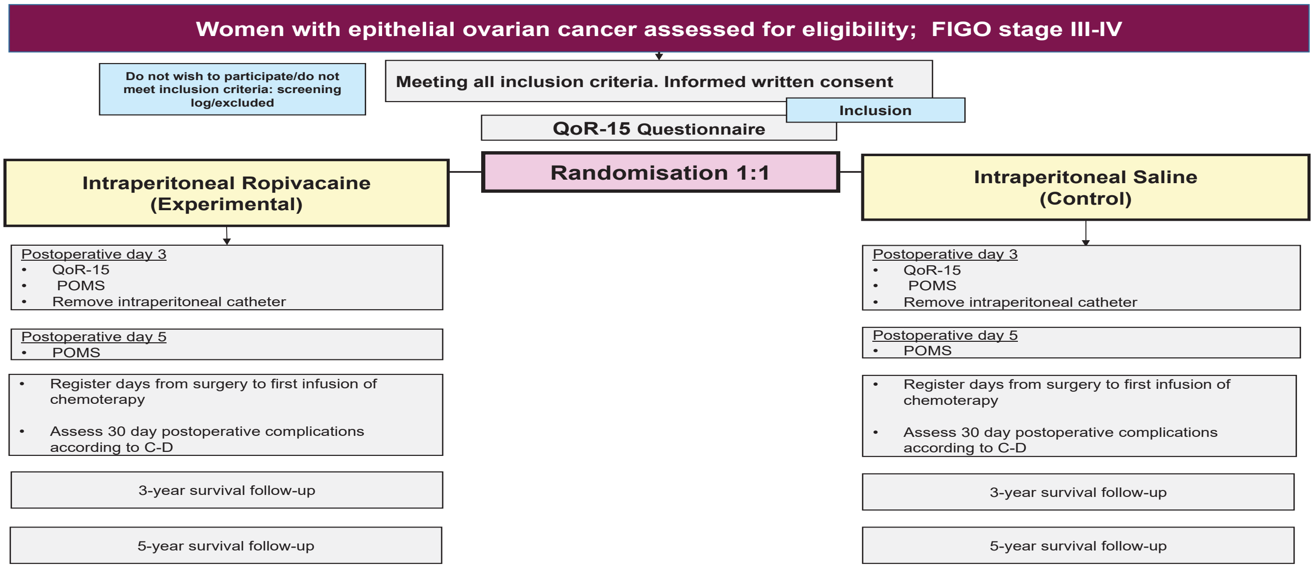

Abbreviations: FIGO, International Federation of Obstetrics and Gynecology; QoR-15, Quality of Recovery questionnaire; POMS, Postoperative Morbidity Score; C-D, Clavien Dindo

3.2 Schedule of events table

Procedures	Enrollment/ Baseline	Day of Surgery	Post-operative day 1	Post-operative day 3	Post-operative day 5	Post-operative day 30	Day of first chemotherapy infusion	Day of last chemotherapy infusion	6 months post-operatively	1 year after surgery	3 years after surgery	5 years after surgery
**Informed consent oral and written**	X											
**Inclusion and exclusion criteria**	X											
**Demographics**	X											
**Blood-samples**[1]	X		X	X	X							
**Quality of recovery Swe-15 Questionnaire**[2]	X			X								
**Randomisation**		X										
**Record operative report**		X										
**Treatment according to randomisation**		X	X	X								
**Removal of intraperitoneal catheter**				X								
**Postoperative morbidity score (POMS)**[3]				X	X							
**Pain management and evaluation**				X								
**Record 30-day post-operative complications according to Clavien Dindo and definitive histology**						X						
**Record primary outcome (days from surgery to start of adjuvant chemotherapy)**									X			
**Record adjuvant treatment**									X			
**Record survival**											X	X
**AE/SAE**		X	X	X	X	X						
**Translational part tissue (site Karolinska only)**		X[4]										
**Translational part blood (site Karolinska only)**[5]		X[4]					X[6]	X[6]		X		

Abbreviations: AE, adverse events; SAE, serious adverse events.

[1] Hb (g/L) CRP (mg/L), Troponin T (ng/L), Albumin (g/L), Natrium (mmol/L), Kalium (mmol/L), eGFR, (ml/min/1,73m^2), Creatinine (micromol/L), S-Transtyretin, P-Fibrinogen,S-Prokalcitonin, CA-125 (kE/L), CEA (microg/L, only preop). [2]see Appendix 17.4. [3]see Appendix 17.2.[4]Collected at beginning and end of surgery. [5]40 mL of blood. [6]Before infusion of chemotherapy.

4 Study enrollment

4.1 Screening procedure and participant identification

All women with epithelial ovarian cancer, FIGO stage III-IV can undergo screening for this trial and will be documented in a screening log. After obtaining oral and written informed consent, patients will be registered and randomized. Registration data must be entered to an electronic Case Report Form (eCRF).

4.2 Inclusion criteria

- Women > 18 years
- ASA I-III
- Scheduled for upfront cytoreductive surgery for stage III or IV epithelial ovarian cancer
- Signed written informed consent

4.3 Exclusion criteria

- Contraindication to epidural anesthesia
- Allergy to any component drugs used during epidural or intraperitoneal anesthesia (Ropivacaine, Sufentanil)
- Uncontrolled renal, liver, heart failure or ischemic heart disease
- Speech, language or cognitive difficulties
- Women in whom cytoreductive surgery is not attempted at time of upfront laparotomy due to extent of disease

4.4 Randomization and registration

After verification of eligibility and signed informed written consent, women will be randomised at the morning of surgery to receive either Ropivacaine or saline by equal allocation, 1:1. The randomisation procedure will be pre-stratified for participating centre (permuted block design).

The Clinical Trials Unit at Centre for Clinical Cancer Studies Theme Cancer, Karolinska University Hospital, Stockholm, Sweden will perform the randomization centrally. All inclusion criteria and no exclusion criteria must be met. Inclusion and exclusion criteria are entered into the randomization/registration application, which is a web-based instrument (ALEA). Username and password are required to log in; each investigator authorized to register patients has a personal login user name and password. If all criteria are met, patients are registered, and the allocated patient study number is recorded in the patients' medical file. After randomization, confirmation e-mail will be sent out to the investigator, research nurse and principal investigator. Randomization will be performed by unblinded dedicated nurses not involved in the study in any other aspect. Each participating center

allocates one dedicated nurse (and one back-up nurse) who will be authorized to enter the web- based randomization system. After randomization a unique study number will be generated for each participant. The code list will be retained in a locked area only accessible to the unblinded nurse (-s).

4.5 Blinding and code-breaking

After allocation has been established, the unblinded nurse then prepares, verifies and label the study drugs in a confined room where both Ropivacaine and Placebo are stored. The syringes and bags for infusion are marked with the patient's unique study number generated at time of randomization. After the intervention/placebo has been prepared their original containers must be put in plastic bag without transparency, closed and then discarded. Both intervention and placebo are color and odorless transparent fluids why blinding can be maintained. The bag for intermittent infusion during the following 72 hours after end of the surgical procedure is covered/masked and secured from opening also marked with patient's study number.

The code list for which treatment the patient was allocated to will be retained in a locked area/cupboard by the unblinded study nurses, not accessible by participating observers or other personnel in the operating rooms, wards or by the attending surgeon, anesthesiologist or oncologist. In this way, full blinding will be achieved at all stages until the end of the study (last patient recruited and followed-up until initiation of adjuvant chemotherapy).

Code-breaking is permitted only if there is a serious negative reaction of IMP to the patient and when determination of the IMP is essential for further management of the patient. The principal investigator should be contacted for code breaking. The contact information to the PI is included in the medical records for each randomized patient. The coordinating investigator will maintain a log of all code-breakings that occur during the trial. The reason for breaking the code must be specified.

4.6 Withdrawal criteria

Subjects can discontinue their participation in the study at any time without any consequence to his/her continued treatment. The investigator/sponsor can at any time terminate the study for a subject due to, e.g., unacceptable adverse events/adverse reactions or because the subject does not follow procedures in the study protocol. If the subject discontinues the study, follow-up of this subject will be performed according to the clinic's routine.

4.7 End of study

The study will end when all patients enrolled in trial have been followed for 5 years, died, withdrawn consent or are lost to follow-up. The trial steering committee may end enrolment at any time if it is deemed that this is in the best interest of the patients. The study may be prematurely terminated due to a high number of serious adverse events related to the IMP or if the enrollment process cannot be completed within a reasonable time frame.

The end of the study will be reported to the MPA within 90 days, or 15 days if the study is terminated prematurely. The Investigators will inform participants and ensure that the appropriate follow up is arranged for all involved.

5 Study treatment

5.1 Intervention

Group IPLA: Ropivacaine 0.2%. After opening the peritoneal cavity and before any surgical dissection, 40 ml of the Ropivacaine will be rinsed in all quadrants within the abdominal cavity under direct vision after opening the peritoneum in order to achieve uniform spread. This will be repeated on two further occasions, first half way through the surgical procedure as estimated by the surgeon and again before closing the abdominal cavity. After the procedure is completed, a plastic multi-port catheter will be inserted lateral to the surgical incision and placed in a way that the tip of the catheter is in the pelvic cavity and the opposite end brought out through the abdominal wall, lateral to the skin incision and fixed with dressing. Ten ml of the Ropivacaine 0.2% will be injected intermittently every other hour via an infusion pump (CADD-Solis) via a bacterial filter that will be programmed to administer an intraperitoneal infusion of 10 mL Ropivacaine 2mg/ml as a bolus injection every other hour for 72 h postoperatively. 72 hours after the end of the surgical procedure the intermittent infusion ends and the intraperitoneal catheter is removed. The amount of infused Ropivacaine 2mg/ml by intraperitoneal infusion will be recorded and the infusion bags discarded in a secure fashion.

5.2 Control

Group Control: 0.9% saline will be used instead of Ropivacaine in an identical manner described above.

5.3 Drug preparation

A nurse who is not involved in patient care will prepare the test medications.

During Surgery: Three sterile 50 ml syringes will be given to the operating nurse prior to surgery start. These will contain 40 ml of either Ropivacaine 2 mg/ml or 0.9% saline: Dose 1, 2 or 3, to be administered at the time of opening the peritoneum, in the middle of surgery and before closing the peritoneum respectively as explained below.

Ropivacaine 0.2% or 0.9% saline will be used as intervention. Three syringes will be given to the operating nurse prior to surgery start. These will contain 40 ml each of Ropivacaine 2 mg/ml) or 0.9% saline (depending on randomization group). After opening the peritoneal cavity and before any surgical dissection, 40 ml of the Ropivacaine or 0.9% saline will be injected in different quadrants within the abdominal cavity under direct vision in order to achieve uniform spread within the abdomen (Dose 1), and the same volume will be injected intraperitoneally to cover the surface of the cavity on two further occasions, first half way through the surgical procedure as estimated by the surgeon (Dose 2) and again before closing the peritoneal cavity (Dose 3). At the end of the procedure, a plastic multi-port catheter will be inserted lateral to the surgical incision and placed in a way that the tip of the catheter is in the pelvic cavity and the opposite end brought out through the abdominal wall, lateral to the skin incision and fixed with dressing.

Post-operative management: All women will be managed in a high-dependency post-operative care unit overnight as appropriate. An epidural infusion containing Ropivacaine 2 mg/ml and Sufentanil 1µg/ml will be administered continuously as per hospital routine (usually 4-14 ml/hour for 3 to 5 days after surgery) for pain management. In case of pain not relieved by epidural analgesia, nurse-controlled intravenous morphine or morphine equivalent as per hospital routine (usually 1-2 mg). Additionally, ten ml of the Ropivacaine 0.2% or 0.9% saline (depending on group allotment) will be injected intermittently every other hour through a bacterial filter and via an infusion pump (CADD-Solis) for 72 h postoperatively. The patients will be transferred to the general ward or later rehabilitation until ready for home discharge, which usually occurs on postoperative day 6 - 14, according to hospital routines. Patients will be followed-up via the hospital journal for later assessment of death at 3- or 5 years after surgery according to existing hospital routines.

6 Routine anesthesia and surgery

Anesthesia: All patients will be given premedication and intraoperative anesthesia according to standardized protocol at all the participating hospitals. A thoracic epidural will be inserted usually at the Th9-Th11 level before induction of general anesthesia. Usually, during surgery, 0.5% Ropivacaine 3-5 mL/hours will be used for pain management. Intravenous injection of Propofol and Remifentanil or equivalent will be used for induction of anesthesia Maintenance of anesthesia is a combination of epidural and inhalation anesthesia during the surgical

procedure and supplemented with infusion of Remifentanil or equivalent if needed. To achieve neuromuscular blockade Rocuronium or equivalent will be used and will be monitored with train of four. Mechanical ventilation with oxygen in air will be used. Monitoring will include: arterial radial line, oxygen saturation and ECG monitoring. If needed, goal-directed therapy and invasive monitoring guidance will be used to standardize intraoperative fluid management. Mean arterial pressure will be maintained above 65mmHg. Cardiac index >2.5 or stroke volume variation <15% will be the cut-off for hemodynamic instability and will be treated with fluid substitution. Hypotension will be treated with norepinephrine infusion if needed.

Surgery: Will be performed by as per clinical routine. The study treatment will not interfere with any part of the surgical treatment. If per-operative evaluation excludes the patients from cytoreductive surgery because disease extent and the abdomen is closed without any resection of tumor, the participant is excluded from the IPLA-OVCA trial.

6.1 Assessments and Procedures

See schedule of events table, 3.2 for overview

Enrolment/Baseline

Screening, and patient information: Preoperatively, patients will be screened by the surgeons/anesthesiologists for inclusion and exclusion criteria. If suitable for inclusion, the patients will be given verbal and written information about the study and allowed to ask questions then or prior to randomization. No patient will be included in the study unless written informed consent is obtained. Demographic data will be recorded and routine blood samples drawn. This visit will take an additional 15-30 min and does not imply an extra visit to the hospital but be a part of the Surgical and/or Anesthetic consultation. QoR-15: A questionnaire - QoR-15- will be completed by the patient.

Day of surgery

Randomization: Will be performed on the morning of surgery and drugs (Ropivacaine or normal saline) prepared by personnel who will not participate in the study.

Perioperative recordings will be made on a standardized Case Record Form (CRF) that includes drugs administered and monitoring performed. All side effects, the operative report, complications and SAE during or after surgery will be recorded.

Postoperative day 1

Routine clinical blood-samples will be drawn.

Postoperative day 3

In addition to routine clinical management including blood samples. QoR-15 will be completed by the patient. Postoperative morbidity score (POMS) will be recorded. The intraperitoneal catheter will be extracted. All adverse events and SAE after surgery will be recorded.

Postoperative day 5

In addition to routine clinical management including blood samples POMS and Adverse events will be registered. All adverse events and SAE after surgery will be recorded

Postoperative day 30

Complications including readmissions as assessed by the C-D classification, length of hospital stay, number of days with Epidural cathether will be recorded. All adverse events and SAE after surgery will be recorded. The final histopathology will be recorded.

~ 6 months postoperatively

The primary endpoint time from surgery to start of chemotherapy will be recorded including type and frequency of adjuvant treatment as decided by the double blinded attending medical oncologist. All adverse events and SAE after surgery will be recorded

3-years after surgery

The medical records will be reviewed for vital status. Date of death (if applicable) will be recorded.

5-years after surgery

The medical records will be reviewed for vital status. Date of death (if applicable) will be recorded.

7 Translational research component

The study will include a translational research component at site Karolinska only, which will have its focus on examining peritoneal response to surgical trauma and local anesthetics. Specifically, mesothelial chemotherapy resistance and inflammation, cell-free tumour DNA and inflammation in blood. The translational studies will be performed blinded for the investigators upon prospectively collected biopsies from the peritoneum at time of surgery

and blood (collected at several time-points during the study), see schedule of events table, 3.2.

8 Statistical considerations

8.1 Primary endpoint

Time (days) to start of adjuvant chemotherapy after cytoreductive surgery

8.2 Secondary endpoints

Postoperative complications, postoperative morbidity, quality of recovery, cardiac and renal impairment and overall survival

8.3 Data analyses

The primary endpoint, time to start of adjuvant chemotherapy will be analyzed using a linear regression model including the variable treatment. Results will be presented as the mean difference in days together with 95% confidence intervals and a Wald-test p-value. The significance level in the main analysis is set to 5%. Regression models including different clinical factors will also be estimated to take potential confounding into account. All analyses of primary and secondary outcome will be performed according to the intention-to-treat principle but may also be presented as per-protocol. For safety data, patients will be analyzed according to per-protocol approach.

Differences in complications, morbidity and in cardiac and renal impairment will be tested using Chi-square tests of independence. For dichotomous outcomes result will also be presented as differences in proportions together with 95% confidence intervals. Overall survival time will be calculated from the date of randomization to the date of death. For patients still alive, survival time will be calculated from the date of randomization to the date of last visit. Results from the survival analysis will be graphically presented as Kaplan-Meir curves. Proportional hazards regression models will be used to estimate the effect of the intervention on time to death. Results will be presented as hazard ratios 95% confidence intervals and Wald test p-values.

8.4 Power and sample size

If the mean time to start of chemotherapy with standard treatment is 31 days (SD=12) the study needs to recruit totally 182 patients in order to detect a reduction in time to chemotherapy with 5 days (from 31 to 26 days) in the experimental group, using a two-sided significance level of 5% and a power of 80%. To compensate for a possible 20% drop out rate the target sample size is set to 220 patients.

9 Ethical considerations

9.1 Risk-Benefit considerations

This study will use an additional drug, ropivacaine, administered intraperitoneally during and after surgery in one group of patients. The drug has been used for > 20 years and is a safe, cheap, long-acting local anesthetic that has analgesic and anti-inflammatory characteristics that can be used for the benefit of patients. It is used for local infiltration, as a spinal anesthetic as well as during nerve blocks. Due to the safety profile of ropivacaine, it has almost completely replaced bupivacaine for routine use, except during spinal anesthesia. There is a risk when using ropivacaine in high doses when local anesthetic toxicity may occur. Symptoms of toxicity include numbness around the mouth, metallic taste on the tongue, light-headedness and later epileptiform seizures. Even hypotension and arrythmias may occur if cardiovascular manifestations occur due to toxicity. Luckily, these are rare if the drug is administered correctly and in the prescribed maximum doses. However, if the drug is used in doses < 750 mg/day (as recommended by the manufacturers), the risk of systemic toxicity is negligible. In a previous study in 40 patients measuring plasma concentration of ropivacaine following intraperitoneal injection (Fig 3), we found that free plasma concentration of Ropivacaine (that determines toxicity) were much lower than known toxic concentrations in humans and no patient revealed signs or symptoms of systemic LA toxicity (43). In the present study, we plan to use similar doses as we used in this previous study.

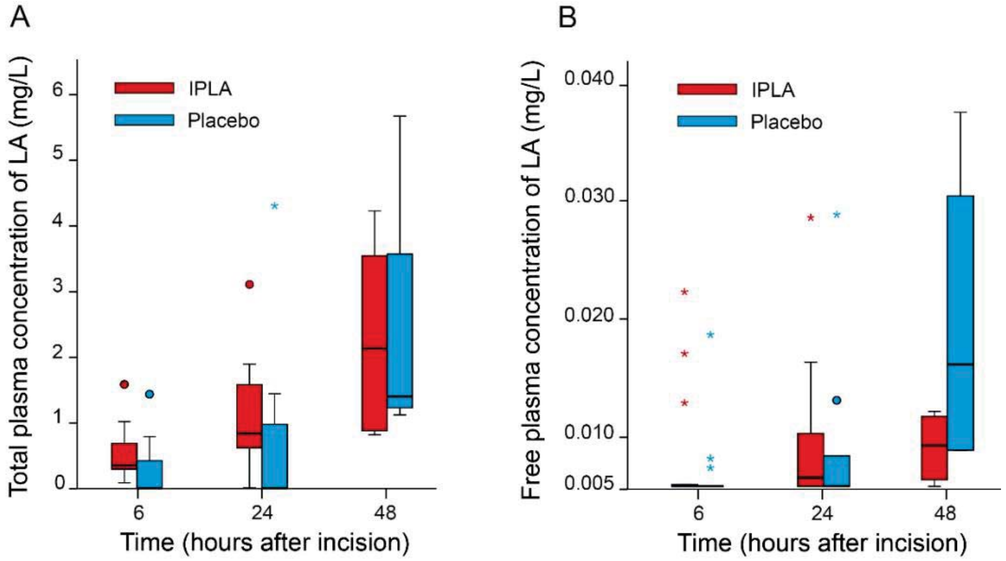

Fig 3. Postoperative serum levels of total and free plasma concentration of Ropivacaine in the two groups at 6, 24 and 48 hours after incision.

There is a potential risk of interaction with opioids and other local anesthetics that may potentiate the effect of ropivacaine. The department is well organized in the potential management of any toxic symptoms and intralipid is easily available and effective.

There is a theoretical risk of catheter-related abdominal infection, however, in our small pilot study we had no catheter related infection. Since the abdominal catheter is inserted under sterile conditions during surgery and a bacterial filter is always used when injecting LA, we do not anticipate any additional risks for participants. However, the patients will be followed-up closely to detect any evidence of infectious complications and start antibiotic management early, if a catheter-related infection is suspected.

In summary, and if our hypothesis is proven correct, it is very much to the advantage for women with advanced EOC subjected to surgery to administer LA, since it may lead to earlier recovery and start of adjuvant chemotherapy. The administration of LA is easy and Ropivacaine is a cheap drug with low toxicity and very few known side effects. The benefits of LA intraperitoneally, if proved, far outweigh the small risk (if any) of systemic toxicity.

9.2 Institutional review board/Ethics committee

The study protocol, patient information and informed consent form will be submitted to the ethics committee for approval. The study will only commence after approval by the ethics committee. All substantial protocol modifications must be submitted to the appropriate Independent Ethics Committee or Institutional Review Board for information and approval before implementation. Once approved by the appropriate Independent Ethics Committee or Institutional Review Board, the investigator shall implement such Protocol modifications. Protocol modifications for urgent safety matters shall however be directly implemented

9.3 Informed consent and withdrawal

Before inclusion in the study, patients will be given oral and written information of the study aims, all treatment procedures and expected and possible adverse events. They will be informed as to the strict confidentiality of their patient data, and that the medical records will be reviewed by their treating physician and study personnel only. The patient is at any time, with or without given reason free to withdraw their consent to study participation, and this choice will not affect their subsequent treatment options or care. Written Informed Consent must be obtained from all participants before enrolment in study. The Informed Consent Form should also be signed at the same occasion by the investigator who gave the written and verbal information. The Informed Consent Form should be filed in the Investigator´s File and one copy should be given to the study participant. The study participants will consent to: participate in the study; regulatory authorities and sponsor’s representative (e.g. monitor) to gain full access to hospital records, to control the data collected in the study; recording,

collecting and processing data and storing data in a database; and storing of study samples in a biobank (if the participating center is part of the translational part of the IPLA-OVCA trial).

9.4 Premature study termination

Premature termination of this clinical study may occur because of a regulatory authority decision or drug safety problems.

If, in the opinion of the Investigator, the clinical observations in the study suggest that it may be unwise to continue the study at the site, the Investigator may terminate participation in the study for ethical, safety, or administrative reasons.

If the study is prematurely terminated, the Investigator should promptly inform the patients and take necessary steps to finalize their engagement in the study. All relevant study material must be collected, and accountability completed.

9.5 Patient protection and Good Clinical Practice

The responsible investigator will ensure that the study is conducted in agreement with the study protocol, ICH-GCP, the declaration of Helsinki and/or Swedish/National laws and regulations; whichever provides the greatest protection for the patient. The participant should be clearly informed that the data collected in the study will not identify any subject taking part in the study following the General Data Protection Regulation (GDPR) (EU 2016/679). Participating women will be treated according to the international guidelines on GCP as defined by the European Parliament (EG596/200).

9.6 Subject identification

Participating patients will be identified by a study specific code consisting of a two to six-digit number. This code will be used when registering the patient into the study database. The woman's national identification number will not be entered into the database. The key to the code will be available to the investigator only.

10 Investigational Medicinal Products

10.1 Description of Investigational Medicinal Products

Reference is made to the SmPCs for further information regarding Ropivacaine. For details about dose and administration see section 5.1-5.3.

The study drugs are provided in full by the investigator. The manufacturer does not supply the pharmacological compound.

10.2 Packaging, Labeling, Storage and Handling of Investigational Medicinal Products

The IMP: s are used according to clinical routine and only the health personnel will dispense the drugs. The study drugs will be kept in a bag with only the study number. The participants and the health care professionals involved in patient care will therefore be fully blinded until the study is completed. Both Ropivacaine 0.2% and saline are colorless and transparent and therefore full blinding will be possible until study completion. The syringes (three per subject) will be labeled with study name, principal investigator, randomization number, who prepared the IMP/placebo, time and date of preparation, signatures of both persons involved in the preparation (please see separate enclosure). The syringes will also be labeled with either of the number 1, 2 or 3 to indicate the order of doses. The bag for infusion will be covered by an additional bag to shield the original labeling, this outer bag will be labeled with study name, principal investigator, randomization number, who prepared the IMP/placebo bag, time and date of preparation, signatures of both persons involved in the preparation.

For further information regarding storage and handling reference is given to the SmPC.

10.3 Placebo

Reference is made to the SmPCs for further information regarding 0.9% saline, which will be handled and labelled in the same way as Ropivacaine.

10.4 Drug accountability

The investigator is responsible for ensuring IMP accountability. The dispensing of the IMP/placebo will be carefully recorded at the site on the appropriate drug accountability forms and an accurate accounting will be available for verification by the monitor at each monitoring visit. IMP accountability records will include:

- The use of each dose by each subject. For injections, the volume injected will be documented as recorded by the infusion pump

- Randomization confirmation

- The disposition of unused IMP

- Dates, quantities, batch numbers, expiry dates and the patient IDs assigned

The investigator should maintain records that adequately document that the IMP was provided to the subjects in the doses specified by the protocol/amendment(s) and randomization.

IMP, which has been dispensed to a subject, must not be re-dispensed to a different subject.

The monitor will periodically control the IMP accountability forms.

10.5 Concomitant Medication

All concomitantly administered medication during the study will be registered including, but not limited to, sedatives and anti-emetics given postoperatively. Prescription and non-prescription medications used at home after discharge and during the study will also be registered.

The investigator should instruct the patient to notify the study site about any new medications he/she takes after the start of the study drug. All medications and significant non-drug therapies (including physical therapy and blood transfusions) administered after the patient starts treatment with study drug must be listed in the CRF and medical records. The patients will record all additional prescription drugs used after home discharge in their diary.

10.6 Rescue treatment

Morphine is administered intravenously as rescue medication to all patients experiencing pain in both groups while in hospital (maximal dose 180 mg/day). Number of doses and amount given Morphine is recorded.

10.7 Continuation of Treatment

No study treatment is planned or anticipated after the end of study. Current hospital routines will be followed in all cases of deviation from normality or in individual patients requiring counselling or other support.

11 Assessment of Efficacy and Safety

11.1 Baseline assessments

A complete medical history will be recorded during the first visit. The medical history will include a review of all past and current relevant diseases and surgeries prior to screening, as judged by the Investigator.

Baseline assessments include assessment of eligibility criteria and registration of current medication. The Investigator or designee will assess changes in concomitant medications throughout the study by asking the patient at each visit. Any changes reported by the patient will be recorded in the CRF.

11.2 Clinical Efficacy Assessments

The following parameters will be recorded:

1. Pain, measured on a numeric rating scale (0-10, where 0 = no pain, 10 = worst imaginable pain). Measured at rest and on mobilization (sitting in bed/walking)

2.Postoperative nausea and vomiting will be registered as yes or no. If yes, whether treated by drugs and which drugs will be registered.

3. Quality of recovery (QoR 15) (appendix 4) assessed preoperatively and after 3 day.

4. Post-operative complications as determined by Clavein-Dindo classification (appendix 2) for surgical complications and Postoperative Morbidity Score (POMS) (appendix 3) during hospital stay.

5. Post-operative morbidity, re-operation, re-admission after primary home discharge during 0-30 days postoperatively.

6. Time to start of first dose of adjuvant chemotherapy will be registered.

7. Overall survival (OS) at 3- and 5-years and time to death will be recorded.

11.3 Clinical Safety Assessments

All side effects (nausea, vomiting, pruritus, respiratory depression, oxygen desaturation and need for supplementary oxygen) and complications related to anesthesia and/or surgery (bleeding, reoperation, pneumothorax etc.) will be recorded during the hospitalization according to standardized methods. Prolonged PACU stay or re-admission to PACU will be registered. Clavien-Dindo classification for grade of complication will be registered as also Postoperative Morbidity score (POMS).

12 Proceedings for Adverse Events

12.1 Definition of Adverse Events

An Adverse Event (AE) is any untoward medical occurrence in a subject administered Investigational Medicinal Product (IMP) and which does not necessarily have a causal relationship with this product. An AE can be any unfavorable and unintended sign, abnormal laboratory finding, symptom or disease temporally associated with the use of IMP, whether or not related to the product. The AE are reported during ongoing study treatment.

12.1.1 Definition of Adverse Reactions

Each AE is to be classified by the investigator as related or not related to the IMP. An Adverse Reaction (AR) is a noxious and unintended medical **response** to a medical product related to any dose. For an AE to be regarded as an AR the suspected association between the product and the unwanted medical condition should be at least a reasonable **possibility**. The AR are reported during ongoing study treatment.

12.1.2 Definition of Serious Adverse Events

Each AE is to be classified by the investigator as serious or non-serious. Seriousness is not defined by a medical term; it is a result or an outcome. An AE is defined as a Serious Adverse Event (SAE) if it:

- results in death
- is life-threatening
- requires inpatient hospitalization or prolongation of existing hospitalization
- results in persistent or significant disability/incapacity
- results in a congenital anomaly/birth defect
- other medically important event

The SAE are reported up to 30 days after end of study treatment (33 days postoperatively)

12.1.3 Definition of Suspected Unexpected Serious Adverse Reactions (SUSAR)

Each SAE that is at least possibly related to an IMP is to be classified by the investigator as expected or unexpected. An SAE that is at least possibly related to an IMP, and **unexpected**, is defined as a Suspected Unexpected Serious Adverse Reaction (SUSAR). It is expected if it is already known from earlier trials or is mentioned in relevant documents (Investigator's Brochure, SPC).

The SUSAR are reported up to 30 days after end of study treatment (33 days postoperatively)

12.2 Assessment of Adverse Events

12.2.1 Assessment of Intensity

Each AE is to be classified by the investigator as mild, moderate or severe.

Mild: Acceptable. The subject is aware of symptoms or signs, but these are easily tolerated.

Moderate: Disturbing. The AE is discomforting enough to interfere with usual daily activities.

Severe: Unacceptable. The subject is incapable of working or performing usual daily activities.

12.2.2 Assessment of Causality

Unlikely: The event is most likely related to a aetiology other than the IMP.

Possible: A causal relationship is conceivable and cannot be dismissed.

Probable: Good reason and sufficient documentation to assume a causal relationship.

12.3 Methods for Eliciting Adverse Events

AEs occurring during the study will be collected at Visits 1-11. All information about the AEs, whether spontaneously reported by the subject, documented in diaries, discovered by the Investigator questioning or detected through physical examination, laboratory test or other means will be documented.

12.4 Adverse Events

12.4.1 Reporting of Adverse Events (AEs)

All AEs will be recorded on a separate AE form in the CRF. The following information will be recorded:

- Description of the event.
- Date of onset.
- Date of resolution (or that the event is ongoing).
- Action taken as a result of the event.
- Seriousness of the event.
- Severity of the event.
- Outcome of the event.
- Investigator´s assessment of relationship to study medication.

Postoperative pain will be documented and analyzed but will not be registered as an AE. Physiological variations related to anesthesia or surgical procedures, postoperative complications, will be recorded but not analyzed as an AE.

12.4.2 Reporting of Serious Adverse Events (SAEs)

In addition, SAEs will also be documented on a separate SAE form within 24 hours after the SAE has been communicated to the investigator. The investigator shall report all serious adverse events immediately, but under no circumstances later than within 24 hours, to the sponsor Follow-up information describing the outcome of the SAE and actions taken will be documented as soon as available. The original SAE form must be filed with the CRF or in the investigator site file. The SAEs will be summarized and analyzed after the study. Scheduled hospitalization or planned surgery for a disease or condition that the patient had prior to inclusion in the study will not be registered as a SAE. SAE will be followed until they are "solved" or until the patient's participation in the study is completed.

12.4.3 Reporting of Suspected Unexpected Serious Adverse reactions (SUSAR)

The sponsor is responsible for registering of all relevant information regarding suspected, unexpected, serious adverse reactions in the EudraVigilance database and for notifying the Ethics committee.

The sponsor is responsible for informing all investigators of SUSARs that have occurred. Reporting of any SUSAR will be done on the CIOMS form that is sent to the Medical Products Agency, as sponsor is not able to report this electronically to the EudraVigilance database.

A SUSAR that resulted in death or was life threatening must be registered promptly, or within 7 days after occurrence or sponsor awareness. Relevant subsequent information should be registered within the following 8 days. All other SUSARs should be reported as soon as possible or within 15 days after sponsor awareness.

12.5 Follow-up of Adverse Events

For all AEs, the subject will be followed until either the AE has ceased or until the subject is under professional medical care and a potential causality between the study treatment and the AE has been assessed.

12.6 Follow-up of post-study survival information

Post study survival information will be collected at 3- and 5-years post study completion for patients that have given their consent.

12.7 Annual Safety Update (DSUR)

As long as the study is ongoing, the sponsor will send an Annual Development and Safety Update Report (DSUR) to the Medical Products Agency and the Ethics committee. The

report includes a summary of all reported SAEs and SUSARs, a summarized safety assessment for study subjects and information regarding potential updates of the risk-benefit assessment since study approval.

12.8 Safety reference information

For reference safety information of the investigational medicinal products, reference is given to the SmPCs. Assessment of an AE/SAE as expected or unexpected will be made with reference to the SmPC.

13 Significance of study

Women with advanced ovarian cancer generally have a dismal prognosis, albeit survival outcomes can be very much improved by radical cytoreductive surgery and chemotherapy combined. However, radical surgery is often extensive with risk of postoperative complications, extended time for recovery and risk of delay in start of adjuvant chemotherapy. If our hypothesis is proven correct it is very much to the advantage for women with advanced EOC subjected to surgery with earlier recovery and start of adjuvant chemotherapy. The administration of LA is easy and Ropivacaine is a cheap drug with low toxicity and very few known side effects. For these reasons, it's use will be implemented as clinical routine.

The IPLA-trials translational part will investigate the impact of surgical trauma on the peritoneal mesothelium by inflammation, circulating tumor cells and chemotherapy resistance. Furthermore, to establish if the anti-inflammatory properties of local anesthetics administered directly in the abdomen also affect chemotherapy resistance, circulating tumour cells which will open novel fields of translational research and the use of anti-inflammatory agents as combination therapy in cancer treatment to prolong survival.

14 Administrative considerations

14.1 Financing

This is an academic study sponsored by the coordinating investigator, Stockholm County Council, with no involvement of any external sponsor. All central administrative expenses related to the trial (statistics, monitoring, questionnaires) are covered by research grants. The goal is to receive enough grants to partially or fully fund the study specific costs for each participating site. Each participating center is free to seek financing of their own

14.2 Publication policy

The results of the study will be submitted for publication in medical journals and will be presented at seminars, congresses and conferences (national and international).

The sponsor will report the study results by to the EudraCT database within 12 months after study termination.

Before publication of the main outcome, no other publication regarding outcome on parts of the cohort can be attempted (except for the translational research component). The Coordinating investigator decides authorship for the main outcome. The members of the trial steering committee must also be (co)-authors in all (other) publications apart from the translation research publications. The Chair of the Sub-committee on translational research will decide authorship on all publications regarding the translational research component, which must include the Coordinating Investigator and member of the Sub-committee. One author (principal investigator) from each participating site, pending data completeness and quality, is to be co-author on any publication reporting on the main findings of the IPLA-trial apart from the translational research findings. If number of authors are limited by the respective scientific journal, contributing sites that have recruited most participants and closed follow up will be selected. All investigators must agree to the fact that upon completion of data collection and analysis of data by study statistician, if the investigators are not in agreement with the outcomes of the results, they may elect to not be part of the authorship of the manuscript; however, the data entered from their site will be maintained and analyzed as agreed at the initiation of the study and confirmed as per of this agreement.

14.3 Adherence to protocol and protocol amendment

The study protocol must be adhered to. Any deviation must be documented, and the Trial steering committee must be informed. Changes or supplements to the study protocol can only be decided on and authorized by the coordination investigator, trial steering committee and statistician. Once approved by the appropriate Independent Ethics Committee or Institutional Review Board, the investigator shall implement such Protocol modifications. Protocol modifications for urgent safety matters shall however be directly implemented.

15 Data management and quality control

15.1 Data source and case report forms

Data collected during the study is pseudonymized so that no individual can be identified. Only persons working in the study will have access to the code list. Clinical data will be entered in a CRF directly from the source documents, which are to be defined at the site

before inclusion of the first subject. In the CRF, the patient is identified only with the study number.

Patient medical records will be source data and will be stored according to Good Clinical Practice (GCP). Data for this study will be recorded, using an electronic Case Report Form (eCRF) and will be transcribed by the site from the paper source documents onto the eCRF. Accurate and reliable data collection will be assured by verification and cross–check of the eCRFs against the investigator's records by the study monitor (source document verification). The study database is situated in Sweden.
The eCRF should be completed for each study subject that has signed the informed consent and has been performing any screening visit procedures. The Investigator is responsible for ensuring the accuracy, completeness, legibility and timelines of the data recorded in the CRFs. The subject's identity must always remain confidential. All subjects will receive a study number , which will be used on the CRF.

15.2 Data recording and record keeping

Data recording and data keeping will be managed by authorized study site personnel designated by the Investigator and stored for a minimum of 10 years after declaration of end of trial. The Investigator and all authorized study site personnel will complete appropriate training prior to the study being initiated and any data being entered into the CRF for any study subject.

15.3 Data protection

Recorded information is confidential, and the database is privacy-protected; i.e., no data can be traced back to the patient in research reports and no unauthorized individuals may have access to the data about individuals in the database. The database will be maintained until further notice (at least 10 years after inclusion of the last patient) and be reported in accordance with the GDPR. The authority responsible for the database is Karolinska University Hospital, Stockholm, Sweden.

15.4 Participant confidentiality

The investigator must assure that patients' anonymity will be maintained and that their identities are protected from unauthorized parties. On CRFs or other documents submitted to the Trial steering committee, patients should not be identified by their names, but with a study number. The investigator should keep a patient enrolment log showing the study number codes, names and addresses.

15.5 Storage of study documents

All original documents such as CRF and study related documents at each site will be stored in fire-safe vaults. To enable audits and evaluations by the Sponsor and inspections by regulatory authorities, the Investigator shall keep records (essential documents) of the study for at least 10 years after end of the study. This includes any original source data related to the study, the subject Identification log (with study numbers, full names and addresses), the original signed Informed Consent Forms and detailed records of investigational products disposition. Patient data that is registered in medical records as part of the routine at each site will only be accessible to the local sites. Pseudonymized data will be sent to the coordinating officer at Karolinska Hospital for central maintenance of all records, but originals will remain at the local sites where patients are recruited.

15.6 Quality Control and Monitoring

To assure that the study is conducted in accordance with the study protocol, that study data is collected properly, that documentation and reporting is done in accordance with ICH-GCP (Good Clinical Practice) and additional ethical and regulatory requirements, the study will be monitored by a qualified professional not involved in the study prior to study start, during, and after study termination. The extent of monitoring will be described in a monitoring plan, based on the sponsor SOPs, protocol and regulatory requirements, which will be approved by the Sponsor. Study conductance, source data, drug accountability, adherence to GCP, and regulatory requirements will be monitored. Monitoring will be performed by experienced personnel at CTO, Centre for Clinical Cancer studies, Theme Cancer, Karolinska University Hospital Solna, which in no way contribute to the actual implementation of the study. There will be separate monitors for blinded and unblinded data respectively, to maintain the double-blinding of the study.

The investigator and other responsible study personnel must be available during the monitor visits and should prepare appropriate material as source data verification documents and CRFs for the monitor. Study personnel should devote enough time to these processes. The investigator will ensure that the clinical investigation participants are aware of and consent that personal information may be scrutinized during the data verification process as part of clinical investigation-related monitoring and auditing by properly authorized persons associated with inspection by domestic and/or foreign regulatory authority (-ies). However, participation and personal information should be treated as strictly confidential to the extent that the applicable law permits and not be publicly available.

15.7 Deviations and Violations

No changes in the study procedures shall be affected without the decision of the Principal Investigator/sponsor. Changes that not classifies as significant may be documented as signed protocol amendments but substantial changes to the assessments or design of the study should be notified for review and approval from the Regulatory Authorities and ethics committee.

Deviations and violations that affect, or potentially may affect the safety of the study subjects or the integrity of the study data are promptly (within 7 days) reported in writing to the MPA.

15.8 Insurance

Study subjects are covered by the patient injury insurance and the Swedish pharmaceutical insurance.

16 Appendix

16.1 Clavien-Dindo

Postoperative complications within 30 days after surgery according to Clavien-Dindo (44)

APPENDIX A. Classification of Surgical Complications

Grades	Definition
**Grade I:**	Any deviation from the normal postoperative course without the need for pharmacological treatment or surgical, endoscopic and radiological interventions.<br>Acceptable therapeutic regimens are: drugs as antiemetics, antipyretics, analgetics, diuretics and electrolytes and physiotherapy. This grade also includes wound infections opened at the bedside.
**Grade II:**	Requiring pharmacological treatment with drugs other than such allowed for grade I complications.<br>Blood transfusions and total parenteral nutrition are also included.
**Grade III:**	Requiring surgical, endoscopic or radiological intervention
**Grade III-a:**	intervention not under general anesthesia
**Grade III-b:**	intervention under general anesthesia
**Grade IV:**	Life-threatening complication (including CNS complications)‡ requiring IC/ICU-management
**Grade IV-a:**	single organ dysfunction (including dialysis)
**Grade IV-b:**	multi organ dysfunction
**Grade V:**	Death of a patient
**Suffix 'd':**	If the patient suffers from a complication at the time of discharge (see examples in Appendix B, http://Links.Lww-.com/SLA/A3), the suffix "d" (for 'disability') is added to the respective grade of complication. This label indicates the need for a follow-up to fully evaluate the complication.

‡ brain hemorrhage, ischemic stroke, subarrachnoidal bleeding, but excluding transient ischemic attacks (TIA); IC: Intermediate care; ICU: Intensive care unit
www.surgicalcomplication.info

16.2 Post-operative morbidity score (POMS)

Post-operative morbidity according to the post-operative morbidity score (45)

**Morbidity type**	**Criteria**	**Source of data**
Pulmonary	Has the patient developed a new requirement for oxygen or respiratory support?	Patient observation<br>Treatment chart
Infectious	Currently on antibiotics and/or has had a temperature of >38°C in the last 24 hr.	Treatment chart<br>Observation chart
Renal	Presence of oliguria <500 mL/24 hr; increased serum creatinine (>30% from preoperative level); urinary catheter in situ.	Fluid balance chart<br>Biochemistry result<br>Patient observation

**Morbidity type**	**Criteria**	**Source of data**
Gastrointestinal	Unable to tolerate an enteral diet for any reason including nausea, vomiting, and abdominal distension (use of antiemetic).	Patient questioning<br>Fluid balance chart<br>Treatment chart
Cardiovascular	Diagnostic tests or therapy within the last 24 hr for any of the following: new myocardial infarction or ischemia, hypotension (requiring fluid therapy >200 mL/hr or pharmacological therapy), atrial or ventricular arrhythmias, cardiogenic pulmonary oedema, thrombotic event (requiring anticoagulation).	Treatment chart<br>Note review
Neurological	New focal neurological deficit, confusion, delirium, or coma.	Note review<br>Patient questioning
Haematological	Requirement for any of the following within the last 24 hr: packed erythrocytes, platelets, fresh-frozen plasma, or cryoprecipitate.	Treatment chart<br>Fluid balance chart
Wound	Wound dehiscence requiring surgical exploration or drainage of pus from the operation wound with or without isolation of organisms.	Note review<br>Pathology result
Pain	New postoperative pain significant enough to require parenteral opioids or regional analgesia.	Treatment chart<br>Patient questioning

16.3 FIGO-staging

2014 FIGO ovarian, fallopian tube, and peritoneal cancer staging system and corresponding TNM (7).

I	Tumor confined to ovaries or fallopian tube(s)	T1
IA	Tumor limited to one ovary (capsule intact) or fallopian tube, No tumor on ovarian or fallopian tube surface No malignant cells in the ascites or peritoneal washings	T1a
IB	Tumor limited to both ovaries (capsules intact) or fallopian tubes<br>No tumor on ovarian or fallopian tube surface<br>No malignant cells in the ascites or peritoneal washings	T1b
IC	Tumor limited to one or both ovaries or fallopian tubes, with any of the following:<br>IC1 Surgical spill intraoperatively<br>IC2 Capsule ruptured before surgery or tumor on ovarian or fallopian tube surface<br>IC3 Malignant cells present in the ascites or peritoneal washings	T1c
II	Tumor involves one or both ovaries or fallopian tubes with pelvic extension (below pelvic brim) or peritoneal cancer (Tp)	T2
IIA	Extension and/or implants on the uterus and/or fallopian tubes/and/or ovaries	T2a
IIB	Extension to other pelvic intraperitoneal tissues	T2b
III	Tumor involves one or both ovaries, or fallopian tubes, or primary peritoneal cancer, with cytologically or histologically confirmed spread to the peritoneum outside the pelvis and/or metastasis to the retroperitoneal lymph nodes	T3
IIIA	Metastasis to the retroperitoneal lymph nodes with or without microscopic peritoneal involvement beyond the pelvis	T1,T2,T3aN1
IIIA1	Positive retroperitoneal lymph nodes only (cytologically or histologically proven)	
IIIA1(i)	Metastasis ≤ 10 mm in greatest dimension (note this is tumor dimension and not lymph node dimension)	T3a/T3aN1
IIIA1(ii)	Metastasis > 10 mm in greatest dimension	
IIIA 2	Microscopic extrapelvic (above the pelvic brim) peritoneal involvement with or without positive retroperitoneal lymph nodes	T3a/T3aN1
IIIB	Macroscopic peritoneal metastases beyond the pelvic brim ≤ 2 cm in greatest dimension, with or without metastasis to the retroperitoneal lymph nodes	T3b/T3bN1
III C	Macroscopic peritoneal metastases beyond the pelvic brim > 2 cm in greatest dimension, with or without metastases to the retroperitoneal nodes (Note 1)	T3c/T3cN1
IV	Distant metastasis excluding peritoneal metastases<br>Stage IV A: Pleural effusion with positive cytology<br>Stage IV B: Metastases to extra-abdominal organs (including inguinal lymph nodes and lymph nodes outside of abdominal cavity) (Note 2)<br>(Note 1: includes extension of tumor to capsule of liver and spleen without parenchymal involvement of either organ)<br>(Note 2: Parenchymal metastases are Stage IV B)	Any T, Any N, M1<br>T3c/T3cN1)

Notes:
1. Includes extension of tumor to capsule of liver and spleen without parenchymal involvement of either organ.
2. Parenchymal metastases are Stage IV B.

16.4 Quality of Recovery, QoR-15

Quality of Recovery Score questionnaire (47).

QoR-15swe (Quality of Recovery-15 Sweden)

Del A
Hur har du mått de senaste 24 timmarna?

(På en skala från 0 till 10, där 0 = Inte någon gång (dåligt) och 10 = hela tiden (utmärkt))

1.	Kunnat andas lugnt	Inte någon gång	0	1	2	3	4	5	6	7	8	9	10	hela tiden
2.	Kunnat njuta av maten	Inte någon gång	0	1	2	3	4	5	6	7	8	9	10	hela tiden
3.	Känt dig utvilad	Inte någon gång	0	1	2	3	4	5	6	7	8	9	10	hela tiden
4.	Kunnat sova gott	Inte någon gång	0	1	2	3	4	5	6	7	8	9	10	hela tiden
5.	Kunnat sköta toalettbesök och personlig hygien utan hjälp	Inte någon gång	0	1	2	3	4	5	6	7	8	9	10	hela tiden
6.	Kunnat kommunicera med anhöriga eller vänner	Inte någon gång	0	1	2	3	4	5	6	7	8	9	10	hela tiden
7.	Fått stöd från sjukhuspersonal	Inte någon gång	0	1	2	3	4	5	6	7	8	9	10	hela tiden
8.	Kan du utföra ditt arbete eller dina vanliga aktiviteter hemma	Inte någon gång	0	1	2	3	4	5	6	7	8	9	10	hela tiden
9.	Känt dig trygg och haft kontroll över din tillvaro	Inte någon gång	0	1	2	3	4	5	6	7	8	9	10	hela tiden
10.	Haft en känsla av allmänt välbefinnande	Inte någon gång	0	1	2	3	4	5	6	7	8	9	10	hela tiden

Del B
Har du känt något av följande symptom de senaste 24 timmarna?

(på en skala från 10 till 0, där 10 = Inte någon gång (utmärkt) och 0 = hela tiden (dåligt))

11.	Medelsvår smärta	Inte någon gång	10	9	8	7	6	5	4	3	2	1	0	hela tiden
12.	Svår smärta	Inte någon gång	10	9	8	7	6	5	4	3	2	1	0	hela tiden
13.	Illamående eller kräkning	Inte någon gång	10	9	8	7	6	5	4	3	2	1	0	hela tiden
14.	Känt ångest eller oro	Inte någon gång	10	9	8	7	6	5	4	3	2	1	0	hela tiden
15.	Känt dig ledsen eller deprimerad	Inte någon gång	10	9	8	7	6	5	4	3	2	1	0	hela tiden

17 References

1. De Angelis R, Sant M, Coleman MP, Francisci S, Baili P, Pierannunzio D, et al. Cancer survival in Europe 1999-2007 by country and age: results of EUROCARE--5-a population-based study. Lancet Oncol. 2014;15(1):23-34.
2. Coburn SB, Bray F, Sherman ME, Trabert B. International patterns and trends in ovarian cancer incidence, overall and by histologic subtype. Int J Cancer. 2017;140(11):2451-60.
3. Bray F, Ferlay J, Soerjomataram I, Siegel RL, Torre LA, Jemal A. Global cancer statistics 2018: GLOBOCAN estimates of incidence and mortality worldwide for 36 cancers in 185 countries. CA Cancer J Clin. 2018;68(6):394-424.
4. Ferlay J SI, Ervik M, Dikshit R, Eser S, Mathers C, Rebelo M, Parkin DM, Forman D, Bray F. GLOBOCAN 2012: Estimated Cancer Incidence, Mortality and Prevalence Worldwide 2012 World Health Organization: International Agency for Research on Cancer; 2012 [Available from: https://publications.iarc.fr/Databases/Iarc-Cancerbases/GLOBOCAN-2012-Estimated-Cancer-Incidence-Mortality-And-Prevalence-Worldwide-In-2012-V1.0-2012.
5. Engholm G, Ferlay J, Christensen N, Bray F, Gjerstorff ML, Klint A, et al. NORDCAN--a Nordic tool for cancer information, planning, quality control and research. Acta oncologica (Stockholm, Sweden). 2010;49(5):725-36.
6. Welfare. TNBoHa. Cancer i siffror 2018. Sweden2018 [Available from: https://www.socialstyrelsen.se/globalassets/sharepoint-dokument/artikelkatalog/statistik/2018-6-10.pdf.
7. Mutch DG, Prat J. 2014 FIGO staging for ovarian, fallopian tube and peritoneal cancer. Gynecol Oncol. 2014;133(3):401-4.
8. samverkan RCi. Statistik Äggstockscancer. Sveriges regionala cancercentrum. Sweden2019.
9. Bookman MA, Brady MF, McGuire WP, Harper PG, Alberts DS, Friedlander M, et al. Evaluation of new platinum-based treatment regimens in advanced-stage ovarian cancer: a Phase III Trial of the Gynecologic Cancer Intergroup. J Clin Oncol. 2009;27(9):1419-25.
10. Di Donato V, Kontopantelis E, Aletti G, Casorelli A, Piacenti I, Bogani G, et al. Trends in Mortality After Primary Cytoreductive Surgery for Ovarian Cancer: A Systematic Review and Metaregression of Randomized Clinical Trials and Observational Studies. Ann Surg Oncol. 2017;24(6):1688-97.
11. Norton L, Simon R. The Norton-Simon hypothesis revisited. Cancer Treat Rep. 1986;70(1):163-9.
12. Bristow RE KB, Chi DS. . Surgery for ovarian cancer principles and practice. New York: Informa Healthcare; 2011.
13. DeVita VT, Jr. The James Ewing lecture. The relationship between tumor mass and resistance to chemotherapy. Implications for surgical adjuvant treatment of cancer. Cancer. 1983;51(7):1209-20.
14. Skipper HE. Adjuvant chemotherapy. Cancer. 1978;41(3):936-40.
15. Heitz F, Harter P, Avall-Lundqvist E, Reuss A, Pautier P, Cormio G, et al. Early tumor regrowth is a contributor to impaired survival in patients with completely resected advanced ovarian cancer. An exploratory analysis of the Intergroup trial AGO-OVAR 12. Gynecol Oncol. 2019;152(2):235-42.
16. Mahner S, Eulenburg C, Staehle A, Wegscheider K, Reuss A, Pujade-Lauraine E, et al. Prognostic impact of the time interval between surgery and chemotherapy in advanced ovarian cancer: analysis of prospective randomised phase III trials. Eur J Cancer. 2013;49(1):142-9.
17. Hofstetter G, Concin N, Braicu I, Chekerov R, Sehouli J, Cadron I, et al. The time interval from surgery to start of chemotherapy significantly impacts prognosis in patients with advanced serous ovarian carcinoma - analysis of patient data in the prospective OVCAD study. Gynecol Oncol. 2013;131(1):15-20.

18. Tewari KS, Java JJ, Eskander RN, Monk BJ, Burger RA. Early initiation of chemotherapy following complete resection of advanced ovarian cancer associated with improved survival: NRG Oncology/Gynecologic Oncology Group study. Ann Oncol. 2016;27(1):114-21.
19. Dunn GP, Old LJ, Schreiber RD. The immunobiology of cancer immunosurveillance and immunoediting. Immunity. 2004;21(2):137-48.
20. Gottschalk A, Sharma S, Ford J, Durieux ME, Tiouririne M. Review article: the role of the perioperative period in recurrence after cancer surgery. Anesth Analg. 2010;110(6):1636-43.
21. Looney M, Doran P, Buggy DJ. Effect of anesthetic technique on serum vascular endothelial growth factor C and transforming growth factor beta in women undergoing anesthesia and surgery for breast cancer. Anesthesiology. 2010;113(5):1118-25.
22. Masoumi Moghaddam S, Amini A, Morris DL, Pourgholami MH. Significance of vascular endothelial growth factor in growth and peritoneal dissemination of ovarian cancer. Cancer Metastasis Rev. 2012;31(1-2):143-62.
23. Yu L, Deng L, Li J, Zhang Y, Hu L. The prognostic value of vascular endothelial growth factor in ovarian cancer: a systematic review and meta-analysis. Gynecol Oncol. 2013;128(2):391-6.
24. Afsharimani B, Cabot P, Parat MO. Morphine and tumor growth and metastasis. Cancer Metastasis Rev. 2011;30(2):225-38.
25. Aghajanian C, Goff B, Nycum LR, Wang YV, Husain A, Blank SV. Final overall survival and safety analysis of OCEANS, a phase 3 trial of chemotherapy with or without bevacizumab in patients with platinum-sensitive recurrent ovarian cancer. Gynecol Oncol. 2015;139(1):10-6.
26. Oza AM, Cook AD, Pfisterer J, Embleton A, Ledermann JA, Pujade-Lauraine E, et al. Standard chemotherapy with or without bevacizumab for women with newly diagnosed ovarian cancer (ICON7): overall survival results of a phase 3 randomised trial. Lancet Oncol. 2015;16(8):928-36.
27. Shi YH, Fang WG. Hypoxia-inducible factor-1 in tumour angiogenesis. World J Gastroenterol. 2004;10(8):1082-7.
28. Masoud GN, Li W. HIF-1alpha pathway: role, regulation and intervention for cancer therapy. Acta Pharm Sin B. 2015;5(5):378-89.
29. Peach G, Kim C, Zacharakis E, Purkayastha S, Ziprin P. Prognostic significance of circulating tumour cells following surgical resection of colorectal cancers: a systematic review. Br J Cancer. 2010;102(9):1327-34.
30. Alkasalias T, Moyano-Galceran L, Arsenian-Henriksson M, Lehti K. Fibroblasts in the Tumor Microenvironment: Shield or Spear? Int J Mol Sci. 2018;19(5).
31. Chamaraux-Tran TN, Mathelin C, Aprahamian M, Joshi GP, Tomasetto C, Diemunsch P, et al. Antitumor Effects of Lidocaine on Human Breast Cancer Cells: An In Vitro and In Vivo Experimental Trial. Anticancer Res. 2018;38(1):95-105.
32. Chamaraux-Tran TN, Piegeler T. The Amide Local Anesthetic Lidocaine in Cancer Surgery-Potential Antimetastatic Effects and Preservation of Immune Cell Function? A Narrative Review. Front Med (Lausanne). 2017;4:235.
33. Piegeler T, Votta-Velis EG, Bakhshi FR, Mao M, Carnegie G, Bonini MG, et al. Endothelial barrier protection by local anesthetics: ropivacaine and lidocaine block tumor necrosis factor-alpha-induced endothelial cell Src activation. Anesthesiology. 2014;120(6):1414-28.
34. Piegeler T, Schlapfer M, Dull RO, Schwartz DE, Borgeat A, Minshall RD, et al. Clinically relevant concentrations of lidocaine and ropivacaine inhibit TNFalpha-induced invasion of lung adenocarcinoma cells in vitro by blocking the activation of Akt and focal adhesion kinase. British journal of anaesthesia. 2015;115(5):784-91.
35. Koltai T. Voltage-gated sodium channel as a target for metastatic risk reduction with re-purposed drugs. F1000Research. 2015;4:297-.
36. Brackenbury WJ. Voltage-gated sodium channels and metastatic disease. Channels. 2012;6(5):352-61.

37. Campbell TM, Main MJ, Fitzgerald EM. Functional expression of the voltage-gated Na(+)-channel Nav1.7 is necessary for EGF-mediated invasion in human non-small cell lung cancer cells. J Cell Sci. 2013;126(Pt 21):4939-49.
38. Diss JK, Stewart D, Pani F, Foster CS, Walker MM, Patel A, et al. A potential novel marker for human prostate cancer: voltage-gated sodium channel expression in vivo. Prostate cancer and prostatic diseases. 2005;8(3):266-73.
39. Gao R, Shen Y, Cai J, Lei M, Wang Z. Expression of voltage-gated sodium channel alpha subunit in human ovarian cancer. Oncology reports. 2010;23(5):1293-9.
40. Schlagenhauff B, Ellwanger U, Breuninger H, Stroebel W, Rassner G, Garbe C. Prognostic impact of the type of anaesthesia used during the excision of primary cutaneous melanoma. Melanoma research. 2000;10(2):165-9.
41. Ramirez MF, Tran P, Cata JP. The effect of clinically therapeutic plasma concentrations of lidocaine on natural killer cell cytotoxicity. Regional anesthesia and pain medicine. 2015;40(1):43-8.
42. Siekmann W TE, Koskela von Sydow A, Gupta A.

The effect of lidocaine and ropivacaine on primary (SW480) and metastatic (SW620) colon cancer cell lines. Oncology Letters 2019.
43. Hayden J, Gupta A, Thorn SE, Thulin P, Block L, Oras J. Does intraperitoneal ropivacaine reduce postoperative inflammation? A prospective, double-blind, placebo-controlled pilot study. Acta Anaesthesiol Scand. 2019;63(8):1048-54.
44. Clavien PA, Barkun J, de Oliveira ML, Vauthey JN, Dindo D, Schulick RD, et al. The Clavien-Dindo classification of surgical complications: five-year experience. Ann Surg. 2009;250(2):187-96.
45. Grocott MP, Browne JP, Van der Meulen J, Matejowsky C, Mutch M, Hamilton MA, et al. The Postoperative Morbidity Survey was validated and used to describe morbidity after major surgery. Journal of clinical epidemiology. 2007;60(9):919-28.
46. Myles PS, Boney O, Botti M, Cyna AM, Gan TJ, Jensen MP, et al. Systematic review and consensus definitions for the Standardised Endpoints in Perioperative Medicine (StEP) initiative: patient comfort. British journal of anaesthesia. 2018;120(4):705-11.
47. Lyckner S, Boregard IL, Zetterlund EL, Chew MS. Validation of the Swedish version of Quality of Recovery score -15: a multicentre, cohort study. Acta Anaesthesiol Scand. 2018;62(7):893-902.
