# Supplementary material for: Effect of intraperitoneal ropivacaine during and after cytoreductive surgery on time-interval to adjuvant chemotherapy in advanced ovarian cancer: a randomised, double-blind phase III trial
Source: Br J Anaesth. 2024 Nov 20;134(3):662–70. doi: 10.1016/j.bja.2024.10.015 (PMC11867074; doi:10.1016/j.bja.2024.10.015)
Supplement: Multimedia component 2 [file mmc2.docx]

| Supplement 2. Additional details on surgical extent, procedures, and outcomes by randomisation arm. | | | |
| --- | --- | --- | --- |
| Variable | **Ropivacaine**  ***n*=86** | **Placebo**  ***n*=89** | **P-value^a^** |
| PCI-score |  |  |  |
| At beginning of surgery |  |  |  |
| Median (IQR) | 21 (11-24) | 17 (13-24) | 0.553^b^ |
| Mean (SD) | 18 (8) | 17 (8) |  |
| At end of surgery |  |  |  |
| Median (IQR) | 0 (0-6) | 0 (0-2) | 0.065^b^ |
| Mean (SD) | 3 (5) | 2 (5) |  |
| Extra-abdominal residual tumour^c^, no. (%) |  |  |  |
| 0 | 80 (93) | 86 (97) | 0.371 |
| 0.1 - 0.5 | 1 (1) | 2 (2) |  |
| 0.6 – 1.0 | 1 (1) | 0 |  |
| >1.0 | 4 (5) | 1 (1) |  |
| Surgical procedures, no. (%) |  |  |  |
| Pelvic procedures | 86 (100) | 86 (97) | 0.246 |
| *Bilateral salpingo-oophorectomy^d^* | *83 (97)* | *80 (90)* |  |
| *Hysterectomy* | *76 (88)* | *75 (84)* |  |
| *Pelvic peritonectomy* | *73 (85)* | *73 (82)* |  |
| *Colorectal resection* | *48 (56)* | *46 (52)* |  |
| *Pelvic nodes* | *14 (16)* | *17 (19)* |  |
| *Partial cystectomy* | *2 (2)* | *1 (1)* |  |
| *Ureteral resection* | *0* | *2 (2)* |  |
|  |  |  |  |
| Medium abdominal procedures | 86 (100) | 89 (100) |  |
| *Peritonectomy gutters* | *62 (72)* | *62 (70)* |  |
| *Small bowel resection* | *12 (14)* | *16 (18)* |  |
| *Small bowel mesentery* | *25 (29)* | *33 (37)* |  |
| *Large bowel resection* | *22 (26)* | *20 (23)* |  |
| *Appendectomy* | *54 (63)* | *52 (58)* |  |
| *Radical omentectomy^e^* | *81 (94)* | *78 (88)* |  |
| *Paraaortic nodes* | *16 (19)* | *11 (12)* |  |
| *Stoma formation* | *24 (28)* | *26 (29)* |  |
| *Defunctioning* | *12 (14)* | *17 (19)* |  |
| *Permanent* | *12 (14)* | *9 (10)* |  |
|  |  |  |  |
| Upper abdominal procedures | 55 (64) | 57 (64) | 1.000 |
| *Diaphragmatic stripping or resection* | *44 (51)* | *48 (54)* |  |
| *Peritonectomy Morrisons pouch* | *32 (37)* | *41 (46)* |  |
| *Resection lesser omentum* | *21 (24)* | *17 (19)* |  |
| *Splenectomy* | *15 (17)* | *16 (18)* |  |
| *Liver capsule resection* | *13 (15)* | *19 (21)* |  |
| *Cardiophrenic nodes* | *4 (5)* | *1 (1)* |  |
| *Hepatic hilum nodes* | *4 (5)* | *1 (1)* |  |
| *Atypical liver resection* | *1 (1)* | *4 (5)* |  |
| *Celiac axis nodes* | *2 (2)* | *3 (3)* |  |
| *Cholecystectomy* | *0* | *3 (3)* |  |
| *Partial gastrectomy* | *0* | *1 (1)* |  |
| *Partial pancreatectomy* | *1 (1)* | *0* |  |

**Abbreviations:** PCI, Peritoneal Cancer Index; IQR, Interquartile range.

**^a^**Fisher’s exact test if not stated otherwise.

**^b^**Mann-Whitney U test. ^c^In centimetres.

**^d^**An additional 4 patients had unilateral salpingo-oophorectomy.

**^e^**An additional 14 patients had infracolic omentectomy.
