# Supplementary material for: Effect of intraperitoneal ropivacaine during and after cytoreductive surgery on time-interval to adjuvant chemotherapy in advanced ovarian cancer: a randomised, double-blind phase III trial
Source: Br J Anaesth. 2024 Nov 20;134(3):662–70. doi: 10.1016/j.bja.2024.10.015 (PMC11867074; doi:10.1016/j.bja.2024.10.015)
Supplement: Multimedia component 3 [file mmc3.docx]

| Supplement 3. Specific postoperative complication within Clavien-Dindo grade $\boldsymbol{\geq}$II, Serious adverse events and volume of study drug. | | |
| --- | --- | --- |
| Variable | Ropivacaine (*n*=86) | Placebo (*n*=89) |
| No complication or grade I, no (%) | 14 (16.3) | 19 (21.3) |
| Specific complication within grade II, no (%)  Pleural fluid  Gastrointestinal complication  Other intestinal complication  Bacterial infection  Blood transfusion  Arrythmia  Embolism  *Missing* | 47 (54.7)  1 (2.1)  2 (4.3)  3 (6.4)  12 (25.5)  26 (55.3)  1 (2.1)  0  *2 (4.3)* | 41 (46.1)  1 (2.4)  1 (2.4)  2 (4.9)  12 (29.3)  22 (53.7)  1 (2.4)  2 (4.9)  *0* |
| Specific complication within grade IIIa, no (%)  Pleural fluid  Pneumothorax  Pancreatic fistula  Gastrointestinal complication  Other intestinal complication  Bacterial infection  Bleeding  Arrythmia  *Missing* | 17 (19.8)  7 (41.2)  1 (5.9)  1 (5.9)  1 (5.9)  4 (23.5)  3 (17.6)  0  0  0 | 23 (25.8)  11 (47.8)  1 (4.3)  1 (4.3)  4 (17.4)  2 (8.7)  1 (4.3)  1 (4.3)  1 (4.3)  *1 (4.3)* |
| Specific complication within grade IIIb, no (%)  Bile leak  Gastrointestinal complication  Abdominal wall complication | 7 (8.1)  1 (14.3)  4 (57.1)  2 (28.6) | 3 (3.4)  0  1 (33.3)  2 (66.7) |
| Specific complication within grade IVa  Arrythmia | 1 (1.2)  1 (100) | 2 (2.2)  2 (100) |
| Specific complication within grade IVb, no (%)  Gastrointestinal complication | 1 (1.2)  1 (100) | 1 (1.1)  1 (100) |
| Grade V, no (%) | 0 | 0 |
| Serious adverse events, no (%)^a^  Grade III  *Arrythmia*  *Superficial wound-rupture requiring intervention*  *Exploratory laparotomy*  *Infection, ileus, pulmonary embolism*  *Post-ERCP pancreatitis*  *Embolization due to bleeding*  Grade IV  *Colorectal anastomotic leakage*  *Leakage transverse colon, sepsis*  *Small bowel injury* | 8 (9)  6 (7)  2 (2)  2 (2)  1 (1)  1 (1)  0  0  2 (2)  1 (50)  0  1 (50) | 6 (7)  4 (5)  2 (2)  0  0  0  1 (1)  1 (1)  2 (2)  1 (50)  1 (50)  0 |
| Total volume of study drug, ml  Median (IQR) | 336 (318-349) | 310 (286-323) |

**Abbreviations:** ERCP, Endoscopic retrograde cholangiopancreatography; IQR Interquartile range.

^a^Events classified as Serious Adverse Events (SAE) were reoperations, need of intensive care, death (all causes) within 33 days of surgery. One grade IV SAE led to premature interruption of intraperitoneal infusion of Ropivacaine. All SAE:s resolved, except one due to atrial fibrillation.
