# Supplementary material for: Effect of intraperitoneal ropivacaine during and after cytoreductive surgery on time-interval to adjuvant chemotherapy in advanced ovarian cancer: a randomised, double-blind phase III trial
Source: Br J Anaesth. 2024 Nov 20;134(3):662–70. doi: 10.1016/j.bja.2024.10.015 (PMC11867074; doi:10.1016/j.bja.2024.10.015)
Supplement: Multimedia component 4 [file mmc4.docx]

| Supplement 4. Postoperative morbidity survey (POMS) postoperative day 3 and 5 by allocation. | | | | | | |
| --- | --- | --- | --- | --- | --- | --- |
|  | **Postoperative day 3** | | | **Postoperative day 5** | | |
| Variable | Ropivacaine  *n*=86 | Placebo  *n*=89 | P-value^a^ | Ropivacaine  *n*=86 | Placebo  *n*=89 | P-value^a^ |
| Any morbidity module, no. (%) |  |  |  |  |  |  |
| Yes | 83 (97) | 82 (92) | 0.169 | 70 (81) | 62 (70) | 0.169 |
| No | 2 (2) | 7 (8) |  | 15 (17) | 25 (28) |  |
| *Missing^b^* | 1 (1) |  |  | 1 (1) | 2 (2) |  |
|  |  |  |  |  |  |  |
| Total number of morbidity module, no. (%) |  |  |  |  |  |  |
| *Median (IQR)* | 3 (2-4) | 3 (2-3) | 0.078^b^ | 2 (1-3) | 2 (0-3) | 0.302^c^ |
|  |  |  |  |  |  |  |
| Morbidity module, no. (%) |  |  |  |  |  |  |
| *Pulmonary* | 38 (45) | 37 (42) | 0.760 | 19 (22) | 20 (23) | 1.000 |
| *Infectious* | 26 (31) | 24 (27) | 0.619 | 26 (31) | 20 (23) | 0.303 |
| *Renal* | 13 (15) | 14 (16) | 1.000 | 10 (12) | 6 (7) | 0.304 |
| *Gastrointestinal* | 25 (29) | 22 (25) | 0.500 | 26 (31) | 31 (36) | 0.520 |
| *Cardiovascular* | 9 (11) | 6 (7) | 0.425 | 6 (7) | 5 (6) | 0.765 |
| *Neurological* | 1 (1) | 0 (0) | 0.489 | 1 (1) | 0 (0) | 0.494 |
| *Haematological* | 19 (22) | 13 (15) | 0.241 | 8 (9) | 8 (9) | 1.000 |
| *Wound* | 2 (2) | 1 (1) | 0.614 | 0 (0) | 0 (0) |  |
| *Pain* | 65 (77) | 61 (69) | 0.309 | 45 (53) | 39 (45) | 0.360 |
| *Mobility* | 59 (69) | 49 (55) | 0.061 | 39 (46) | 35 (40) | 0.538 |
| Abbreviations: IQR, Interquartile range. ^a^Fisher exact test if not stated otherwise. ^b^ 1 patient was discharge before postoperative day 5 and 2 patients had missing data.  ^c^Mann-Whitney U test. | | | | | | |
